# Performing group-level functional image analyses based on homologous functional regions mapped in individuals

**DOI:** 10.1371/journal.pbio.2007032

**Published:** 2019-03-25

**Authors:** Meiling Li, Danhong Wang, Jianxun Ren, Georg Langs, Sophia Stoecklein, Brian P. Brennan, Jie Lu, Huafu Chen, Hesheng Liu

**Affiliations:** 1 Key Laboratory for NeuroInformation of Ministry of Education, School of Life Science and Technology, University of Electronic Science and Technology of China, Chengdu, China; 2 Athinoula A. Martinos Center for Biomedical Imaging, Department of Radiology, Massachusetts General Hospital, Harvard Medical School, Charlestown, Massachusetts, United States of America; 3 National Engineering Laboratory for Neuromodulation, School of Aerospace Engineering, Tsinghua University, Beijing, China; 4 Department of Biomedical Imaging and Image-guided Therapy, Computational Imaging Research Lab, Medical University of Vienna, Vienna, Austria; 5 Institute of Clinical Radiology, Ludwig-Maximilians University of Munich, Munich Germany; 6 McLean Hospital, Harvard Medical School, Belmont, Massachusetts, United States of America; 7 Department of Radiology, Xuanwu Hospital, Beijing, China; 8 Beijing Institute for Brain Disorders, Capital Medical University, Beijing, China; University of Oxford, United Kingdom of Great Britain and Northern Ireland

## Abstract

Functional MRI (fMRI) studies have traditionally relied on intersubject normalization based on global brain morphology, which cannot establish proper functional correspondence between subjects due to substantial intersubject variability in functional organization. Here, we reliably identified a set of discrete, homologous functional regions in individuals to improve intersubject alignment of fMRI data. These functional regions demonstrated marked intersubject variability in size, position, and connectivity. We found that previously reported intersubject variability in functional connectivity maps could be partially explained by variability in size and position of the functional regions. Importantly, individual differences in network topography are associated with individual differences in task-evoked activations, suggesting that these individually specified regions may serve as the “localizer” to improve the alignment of task-fMRI data. We demonstrated that aligning task-fMRI data using the regions derived from resting state fMRI may lead to increased statistical power of task-fMRI analyses. In addition, resting state functional connectivity among these homologous regions is able to capture the idiosyncrasies of subjects and better predict fluid intelligence (gF) than connectivity measures derived from group-level brain atlases. Critically, we showed that not only the connectivity but also the size and position of functional regions are related to human behavior. Collectively, these findings suggest that identifying homologous functional regions across individuals can benefit a wide range of studies in the investigation of connectivity, task activation, and brain-behavior associations.

## Author summary

No two individuals are alike. The size, shape, position, and connectivity patterns of brain functional regions can vary drastically between individuals. While interindividual differences in functional organization are well recognized, to date, standard procedures for functional neuroimaging research still rely on aligning different subjects’ data to a nominal “average” brain based on global brain morphology. We developed an approach to reliably identify homologous functional regions in each individual and demonstrated that aligning data based on these homologous functional regions can significantly improve the study of resting state functional connectivity, task-fMRI activations, and brain-behavior associations. Moreover, we showed that individual differences in size, position, and connectivity of brain functional regions are dissociable, and each can provide nonredundant information in explaining human behavior.

## Introduction

In functional MRI (fMRI) studies, comparing functional characteristics between subjects or groups requires aligning the individual’s data to an “average brain” based on global brain morphology [1]. It is becoming increasingly recognized that interindividual variability exists not only in macroscopic and microscopic brain anatomy [2–4] but also in the organization of functional systems; i.e., the size, shape, position, and connectivity profile of the functional regions may vary drastically across individuals [5,6]. Standard procedures for cross-subject alignment according to macroscopic anatomy may not establish the proper functional correspondence between subjects and can obscure biologically important signals both at the subject level and the group level, especially in the heteromodal association networks that are not strongly tied to anatomical structures [7,8]. For example, one of the best-studied function-anatomy dissociations is in the language network, which may be dominated either by the left hemisphere or by the right hemisphere in different subjects [9–11]. More generally, the high level of intersubject variability in the association functions may be a fundamental principle of brain organization and a critical outcome of human brain evolution [5 6,12]. Recognizing the significance of intersubject variability in functional organization [13,14], the field of neuroimaging has been making rapid progress towards mapping functional regions at the level of individual subjects [15–20], especially using connectivity measured by resting state fMRI. For example, Hacker and colleagues proposed an artificial neural network to localize the motor cortex in individual subjects [21]; integrating information derived from functional and anatomical imaging, Glasser and colleagues proposed a multimodal approach to parcellate the cerebral cortex into hundreds of areas [22]. We recently developed an iterative parcellation procedure to map the individual subject’s cortical functional networks and demonstrated that the results were comparable to the current gold standard, invasive cortical stimulation mapping, in patients undergoing brain surgery [17]. Focusing on subject-level analyses, Gordon and colleagues [19] and Braga and colleagues [23] carefully examined a few subjects who were densely sampled and discovered important features of brain networks that were missed in group-based templates but are evident within the individuals. These technical advances in subject-level functional mapping will not only facilitate the investigation of within-subject functional dynamics [24] that are necessary for personalized medicine [25–27] but will also benefit traditional group-level functional studies by providing more meaningful landmarks for between-subject comparison. Specifically, aligning subjects based on homologous functional regions is expected to improve the specificity of functional signals in the networks being studied, and will lead to increased statistical power in group-level analyses [22,28]. Here, we tested these hypotheses using large-scale resting state and task-fMRI data provided by the Human Connectome Project (HCP) [29–31], and systematically examined how the individually specified functional regions may benefit group-level studies of functional connectivity and task-evoked activations, and in turn facilitate the discovery of brain-behavior associations.

## Results

### Identifying common functional regions across individuals

**Fig 1:**
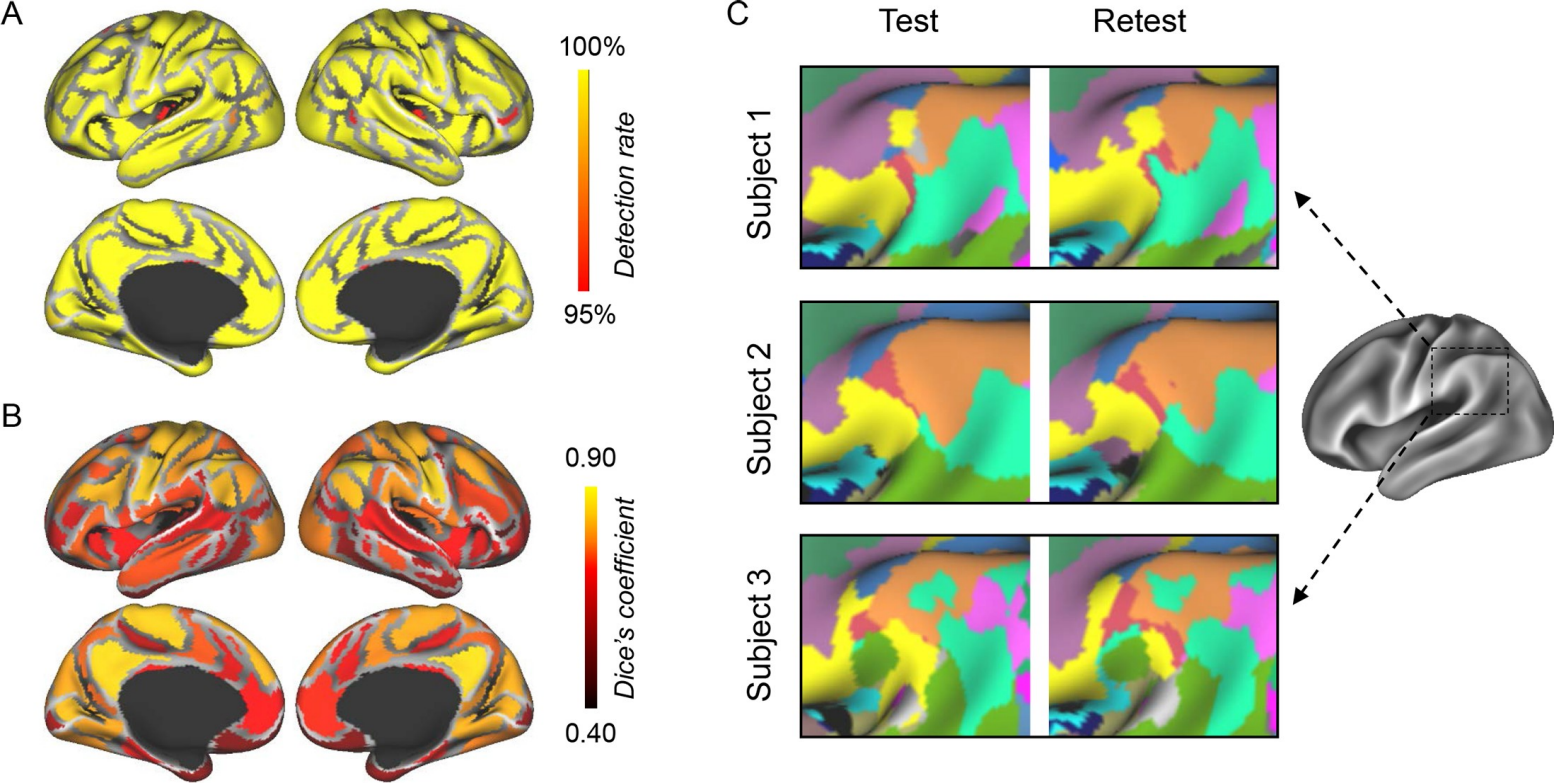
Homologous functional ROIs can be identified across subjects. (**A**) Most of the 116 functional ROIs originally defined in the group-level atlas can be identified in individual subjects. The map demonstrates the percentage of the 677 subjects in whom a functional ROI could be detected. A few ROIs that were not identified in all subjects tended to be smaller in size (shown in red and orange). (**B**) Within-subject test-retest reliability values of the 116 functional ROIs. The test-retest reliability of each ROI was measured as the Dice’s coefficient of the results derived from the two scan sessions of each subject and then averaged across the 677 subjects. For each subject, if a region was undetected in one session, then the Dice’s coefficient of this region was set to zero. The mean Dice’s coefficient across the 116 ROIs was 69.8% ± 12.9% (mean ± SD). (**C**) ROIs in the TPJ extracted from three randomly selected subjects are illustrated as examples. The TPJ region is shown because it consists of multiple small patches belonging to different functional networks. The ROIs were reliably identified across different scan sessions. Different ROIs are represented by different colors. ROI, region of interest; TPJ, temporal-parietal junction.

Using a subject-specific, iterative functional network parcellation strategy that was guided by a group-level functional atlas (Yeo’s atlas) [17,32], we mapped 18 cortical networks for each of the 677 subjects provided by the HCP (selected from the HCP S900 release; see Materials and methods for subject inclusion criteria). The resulting cortical networks were then compared with the initial group-level atlas, which consisted of 116 discrete regions of interest (ROIs) across the 18 cortical networks. Using a template matching approach, we localized the homologous ROIs in each individual subject’s cortical networks (see Materials and methods and S1 Fig). A majority of the 116 ROIs could be identified in each individual. Across the 677 subjects, 92 homologous ROIs were found in all subjects and these ROIs covered 85.6% ± 1.5% of the cortical area (Fig 1A). A few small ROIs were not detected in all individuals, either due to technical limitations or because some functional regions may be truly absent in some subjects [15]. Functional ROIs extracted from the individuals’ networks also demonstrated good reproducibility across different days. Using the data of two scan sessions from the same individual, test-retest reliability was quantified for each ROI. The mean Dice’s coefficient across all ROIs was 69.8% ± 12.9% (Fig 1B). Test-retest reliability was relatively low for the ROIs in the basal frontal and temporal regions, which are more prone to MRI susceptibility artifacts. Importantly, the individually specified ROIs exhibited substantial intersubject variability in size, shape, and position (Fig 1C). These homologous ROIs established a one-to-one correspondence between subjects and thus could be used as the basis for between-subject comparisons in functional studies.

### Revealing intersubject variability in the size, position, and connectivity of the functional regions

Previous studies have repeatedly demonstrated that functional connectivity is highly variable across individuals, especially in the higher-order association areas [5,33], and that the connectivity variability could be related to individual differences in cognitive ability or behavior. However, intersubject variability in connectivity was estimated after aligning data based on brain anatomy; thus, it was influenced both by variability in network topography and variability in connectivity strength among functional regions. Localizing homologous regions in individual subjects allows one to directly evaluate intersubject variability in the size, position, and connectivity strength of the functional regions and investigate their potential relationship to behavior, respectively.

**Fig 2:**
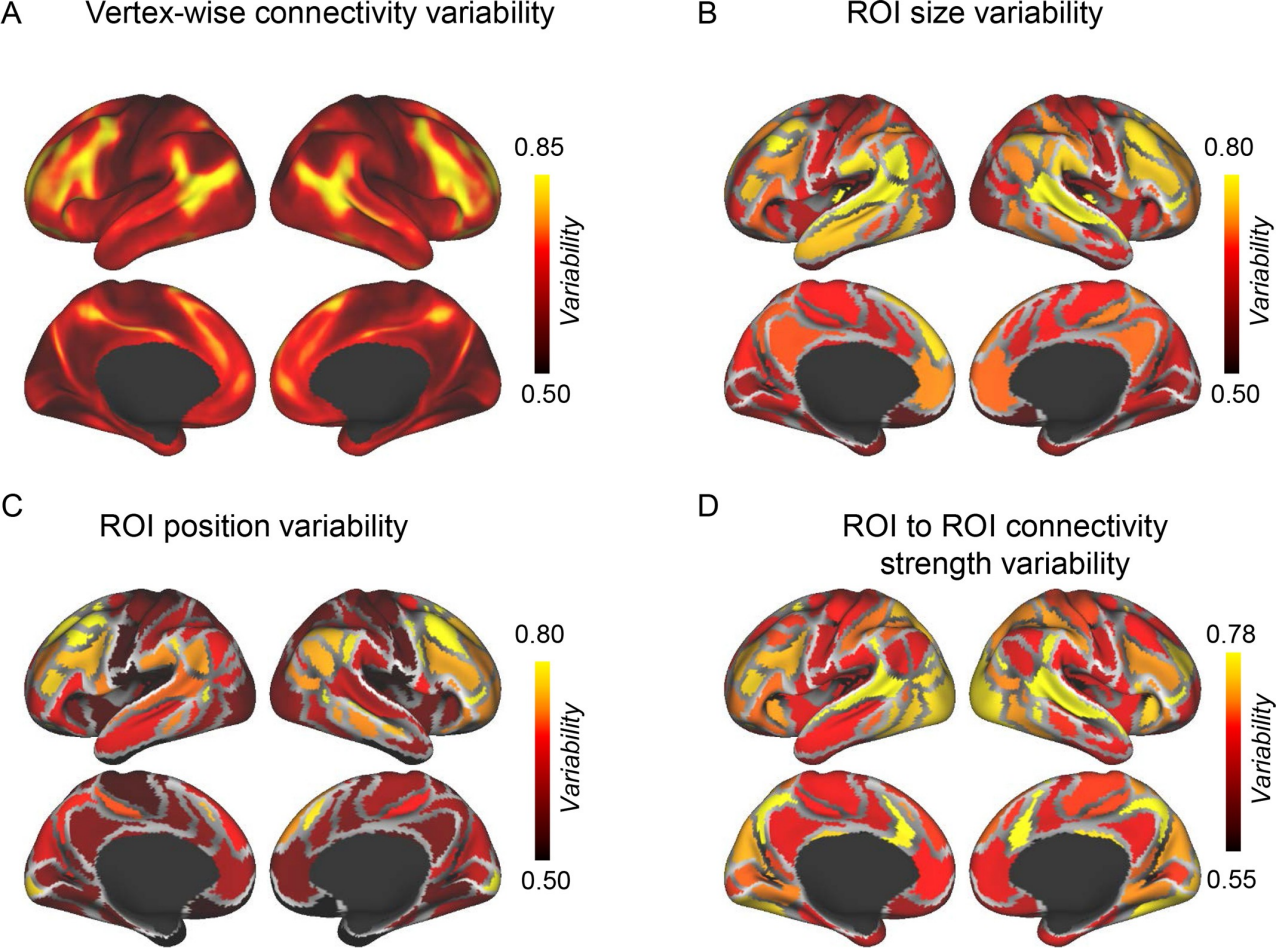
Intersubject variability in vertex-wise functional connectivity maps is associated with intersubject variability in the position of the functional regions. (**A**) Intersubject variability in resting state functional connectivity was quantified at each vertex using the approach as described in Mueller and colleagues [5]. The association cortices showed stronger intersubject variability than the visual and motor-sensory cortices. (**B**) Intersubject variability in ROI size was quantified for each of the 116 ROIs, and the variability map showed a moderate correlation (*r* = 0.26) with the variability in vertex-wise connectivity. (**C**) Intersubject variability in ROI position was quantified for each ROI and showed a strong correlation (*r* = 0.49) with the variability in vertex-wise connectivity. (**D**) Intersubject variability in connectivity among individually specified ROIs was quantified and showed a strong correlation (*r* = 0.55) with the variability in vertex-wise connectivity (also see S2 Fig for the scatterplots). The visual and auditory cortices demonstrated unexpectedly strong intersubject variability in connectivity with other ROIs. All intersubject variability maps were corrected by underlying intrasubject variability (see Materials and methods). ROI, region of interest.

We first quantified intersubject variability in vertex-based functional connectivity maps across the 677 subjects using the approach established in previous studies [5] and successfully replicated the earlier findings (Fig 2A). Intersubject variability in size and position of the 116 ROIs, as well as connectivity among the individually specified ROIs, was then evaluated (Fig 2B–2D, see Materials and methods). We found that intersubject variability in vertex-based functional connectivity maps was not only associated with the variability in connectivity strength among the ROIs (*r* = 0.55, *p* < 0.001, S2 Fig) but was also associated with intersubject variability in position and size of the ROIs (*r* = 0.49, *p* < 0.001 and *r* = 0.26, *p* < 0.005, respectively). These results indicated that previous findings of individual differences in connectivity were likely influenced by variability in network topography. Intriguingly, when variability in functional anatomy was controlled, functional ROIs in the visual and auditory cortices demonstrated strong intersubject variability in their connectivity strength with other ROIs (Fig 2D), although, traditionally, these primary functions were considered less variable. Thus, while visual and auditory ROIs showed relatively low intersubject variability in position (Fig 2B and 2C), which is consistent with previous knowledge that visual and auditory functions are strongly tied to anatomical structures, their connectivity profiles may be significantly more variable across individuals than previously thought (Fig 2D). Taken together, our findings indicated that the size, position, and connectivity of functional regions are dissociable and may be related to different aspects of individual variability in brain functions.

### Individual differences in functional anatomy predict individual differences in task-evoked activations

**Fig 3:**
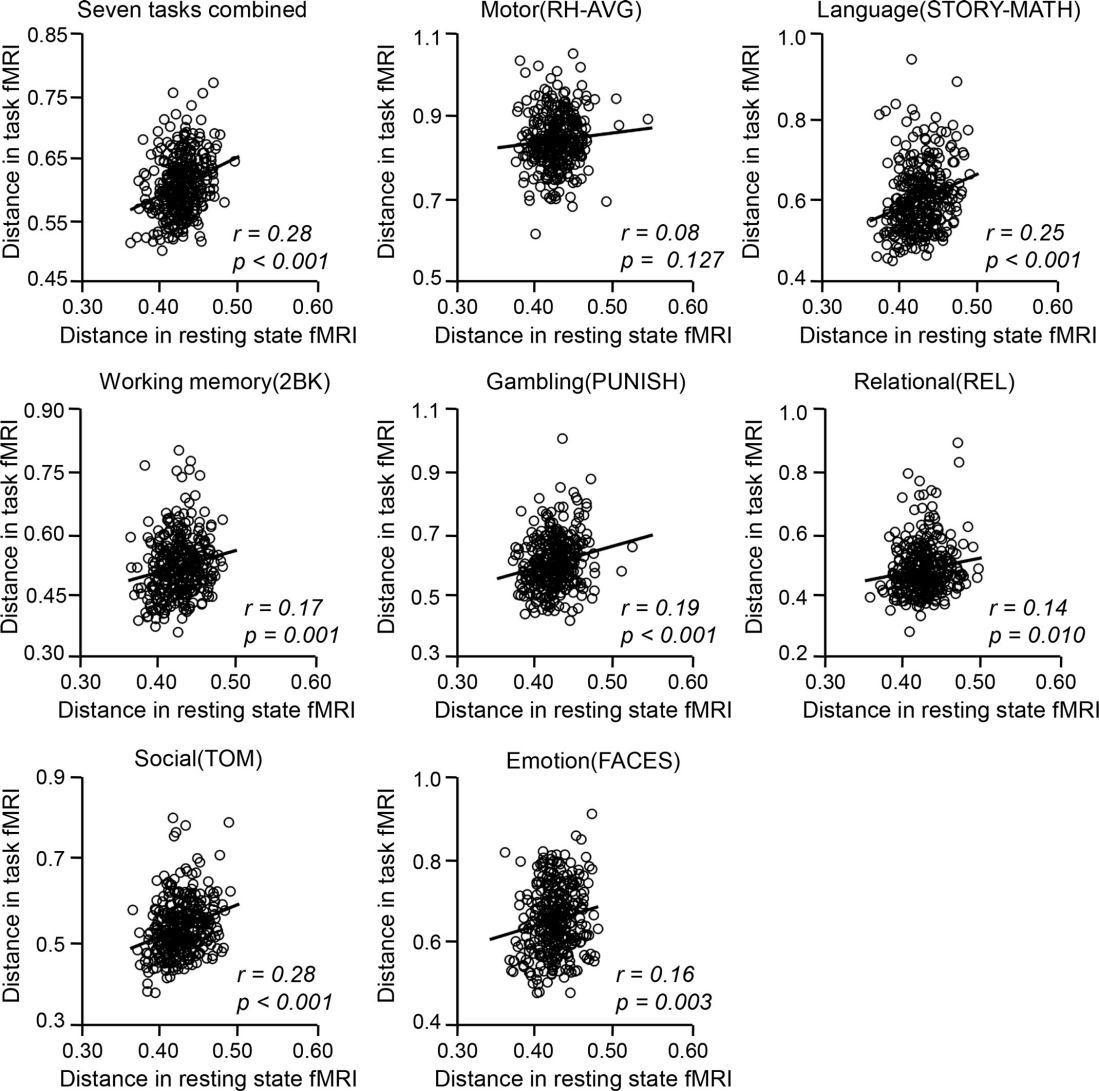
Intersubject variability in cortical functional anatomy is associated with intersubject variability in task-evoked activations. Intersubject variability in cortical functional anatomy was computed as the “distance” between two subjects’ functional ROI distributions (i.e., 1—Dice’s coefficient). Similarly, intersubject variability in task-evoked activations was measured as the “distance” between two subjects’ cortical activation patterns (i.e., 1—spatial correlation between the two maps). Intersubject variability in cortical functional anatomy showed significant correlations with intersubject variability in task activation maps for 6 tasks except for the motor task (*r* values are displayed in each plot, Pearson’s correlation). Each circle represents the “distance” between a pair of subjects; 338 independent pairs of subjects were randomly selected 99 times and the scatterplot of the median performance (correlation) is shown. See S1 Data for numerical values. fMRI, functional MRI.

Spontaneous brain activity and task-evoked activity are bound by the same anatomical connectivity infrastructure; however, the exact relation between resting state connectivity and task-evoked activations remains unclear. Here, we examined whether individual differences in the cortical functional anatomy are related to individual differences in task activation patterns. Intersubject variability in cortical functional anatomy was computed according to the Dice’s overlap between two subjects’ functional ROI distributions (i.e., 1—Dice’s coefficient, “rest distance”). Task-evoked activity was first estimated on the cortical surface of each individual (i.e., beta values derived from the general linear model); intersubject variability was then measured as the “distance” between two subjects’ cortical activation patterns (i.e., 1—spatial correlation between the two task-fMRI maps, “task distance”). We found that intersubject variability in cortical functional anatomy was significantly associated with intersubject variability in task-evoked activations (Fig 3, *r* = 0.28, *p* < 0.001 for seven tasks combined and *p* < 0.05 for all individual tasks except the motor task). In other words, subjects who showed similar cortical distribution of the functional regions tended to show similar task activation maps. These findings provided a theoretical ground for using the ROIs derived from resting state functional connectivity for task-fMRI studies.

### Aligning functional regions across individual subjects improves group-level task-fMRI statistics

**Fig 4:**
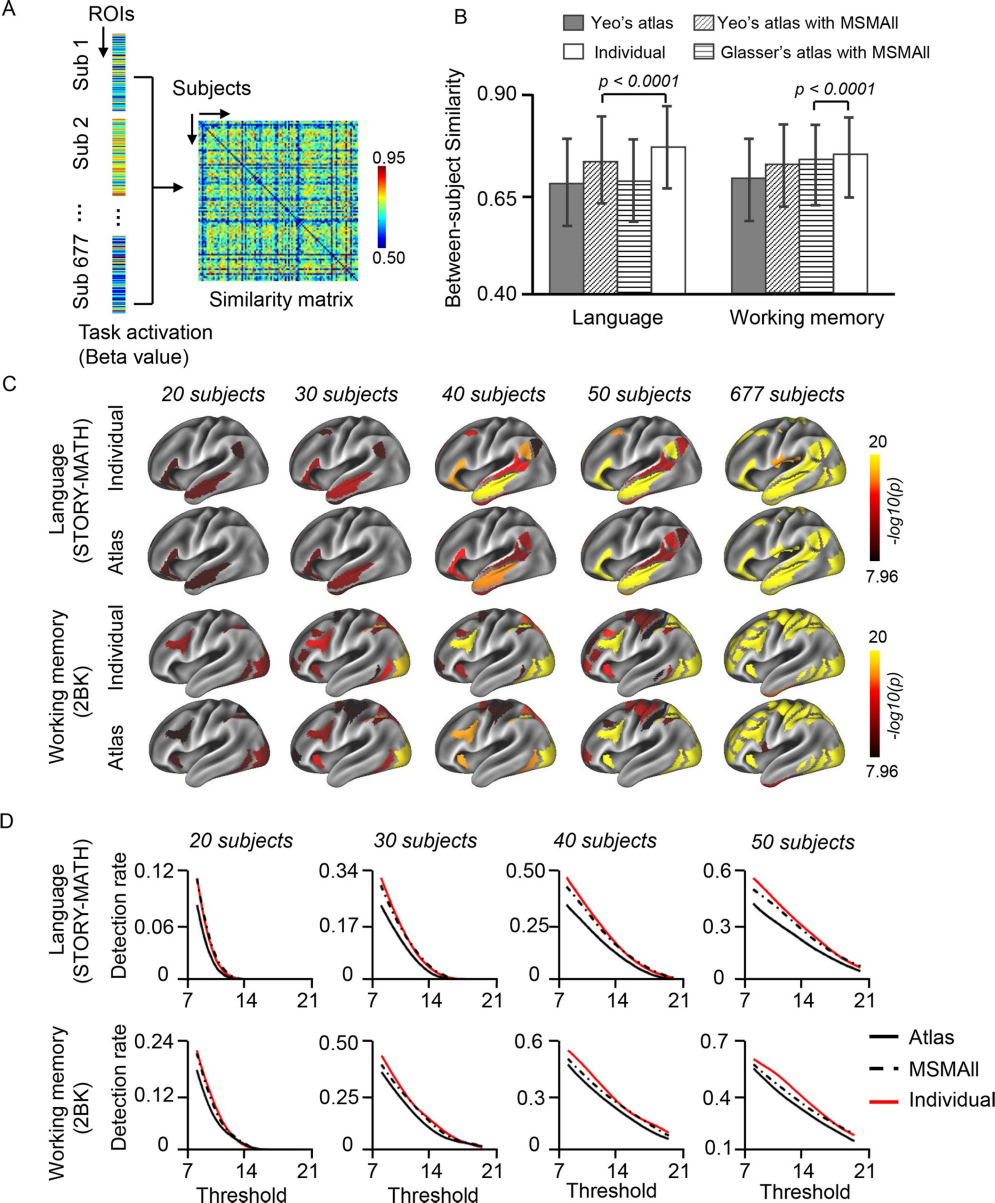
Aligning functional regions across individual subjects improves group-level task-fMRI analyses. (**A**) Similarity of task activations between pairs of subjects. For each subject, task activation values (beta values) were averaged within each ROI; thus, the whole-brain activation pattern was represented by activations in ROIs. Similarity between two subjects was estimated by correlating their activation pattern in these ROIs. (**B**) The bar plots demonstrate the mean between-subject similarity values during the language and working memory tasks estimated by different approaches. Task activation patterns were more similar between two subjects if the ROIs were individually specified compared with atlas based (whether or not the data were aligned by MSMAll) (*p < 0.001, block bootstrap test, 1,000 permutations). Error bars indicate 2 standard deviations. See S3 Fig for the results of other tasks. (**C**) Group-level statistical analyses were performed in the individually specified ROIs and atlas-based ROIs using the mean activation (beta values) within each ROI (one-sample *t* test, *p* < 0.000001 for language and working memory tasks, Bonferroni correction for 92 comparisons). Results of the language and working memory tasks in subsets of the cohort (*n* = 20, 30, 40, 50) and in the full cohort were plotted (see S3 Fig for the results of other tasks). (**D**) Task-relevant regions could be better detected using the individually specified ROIs than using the atlas-based ROIs, independent of the selection of a significance threshold. Group-level task-activated regions were mapped using a series of significance thresholds. The results were then compared with the task-activated regions identified in the full cohort to determine the detection rate. The detection rate was higher for individually specified ROIs (red curves) than atlas-based ROIs (black curves). The MSMAll (dashed curves) improved the task activation but not as much as the individual ROIs. See S1 Data for numerical values. fMRI, functional MRI; MSM, multimodal surface matching; ROI, region of interest; Sub, subject.

We examined whether task-evoked activity could be better aligned across individuals using the individually specified functional ROIs than using the atlas-based ROIs. Task activation maps were first estimated on the cortical surface of each individual using the general linear model, and then activation values (beta values) were averaged within each ROI. A subject’s whole-brain activation pattern was thus represented by the activation values in these ROIs. Similarity between two subjects was estimated by correlating their activation patterns (Fig 4A). We found that task-evoked activation patterns were significantly more similar between two subjects when activations were estimated using our individually specified ROIs, as opposed to using the ROIs from group-level atlases including Yeo’s atlas [32] and Glasser’s atlas [22]. This remained true when the data were processed using MSMAll, an improved cross-subject registration approach based on a multimodal surface matching (MSM) algorithm and some useful imaging modalities released by the HCP [28,34] (*p* < 0.001 for 5 of the 6 tasks in the HCP data, block bootstrap test accounted for the family structure, 1,000 iterations. See Fig 4B for the Language task and Working Memory task as examples. See S3 Fig for the results of the other tasks).

Improved cross-subject alignment may result in an increased statistical power in group-level task-fMRI analyses. To test this hypothesis, a group-level one-sample *t* test was performed using the individuals’ mean activation values (beta values) within each ROI. The task-relevant regions were first identified using the full sample of 677 subjects. Group-level statistical analyses were then carried out using subsets (20, 30, 40, 50 subjects) of the cohort, and the regions showing significant activations were plotted to the brain surface (see Fig 4C for the results of the Language and Working Memory tasks as examples). In these subsets of subjects, activations were more significant when the analyses were performed using the individually specified ROIs than the atlas-based ROIs. Task-activated regions were also mapped using a series of significance thresholds and compared with the maps derived from the full dataset of 677 subjects. In these subsets of subjects, a higher percentage of task-relevant regions could be detected when the group-level statistical analyses were carried out using the individually specified ROIs than the atlas-based ROIs, across different selections of significance thresholds (Fig 4D, see S3 Fig for results of other tasks). Finally, we found MSMAll processing could improve cross-subject alignment but did not outperform our approach based on individually specified ROIs (Fig 4D).

### Functional connectivity among the individually specified ROIs better predicts fluid intelligence than connectivity among the atlas-based ROIs

**Fig 5:**
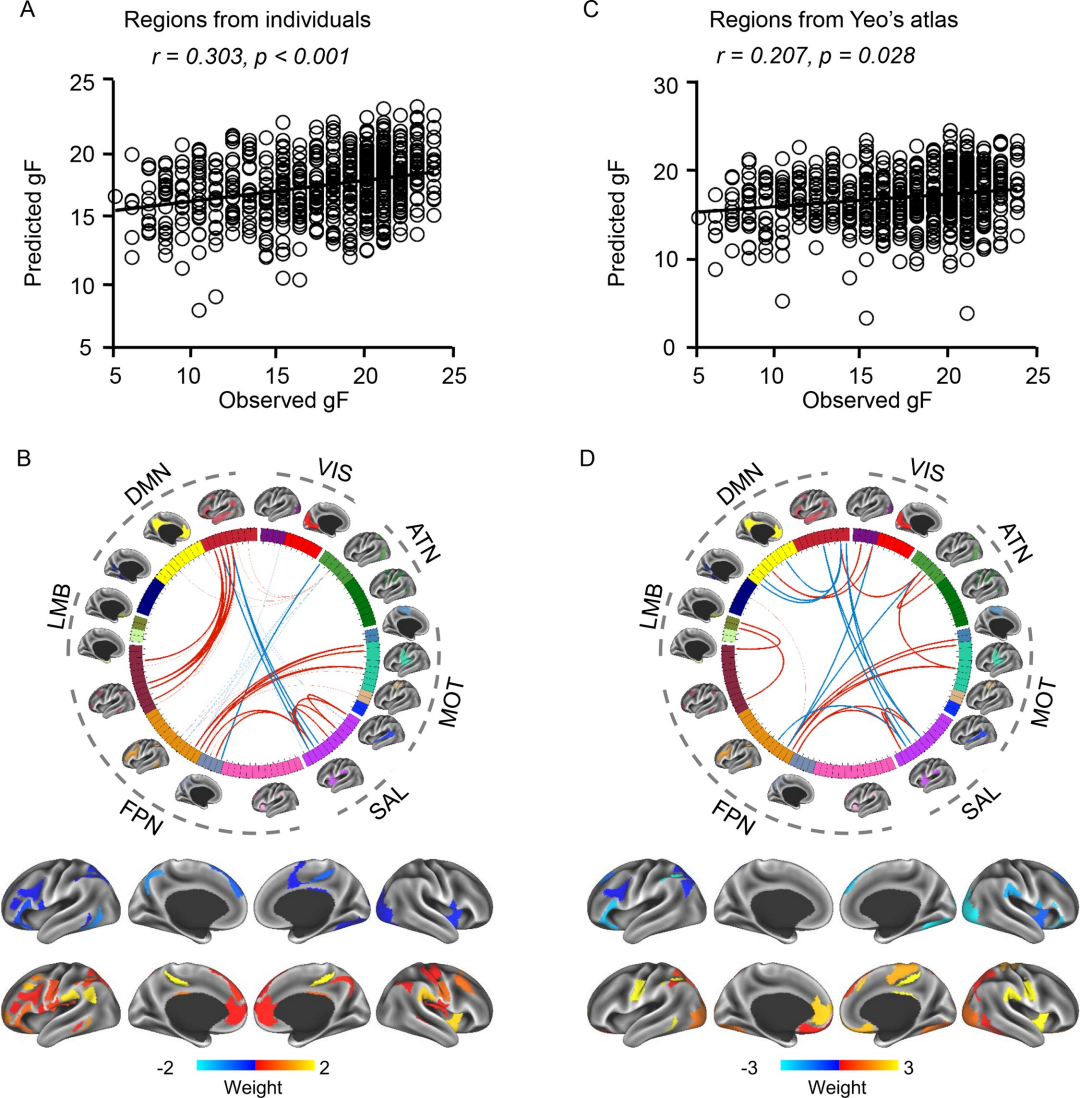
Functional connectivity among the individually specified ROIs can better predict gF than connectivity among the atlas-based ROIs. (**A**) gF was predicted based on connectivity values among the individually specified ROIs. The scatterplot demonstrates the correlation between the predicted and observed gF scores (Pearson’s correlation, *r* = 0.303, *p* < 0.001). Each circle represents a subject. Correlation significance was determined using 1,000 permutations. (**B**) ROI pairs contributing to the prediction. Ninety-two homologous ROIs extracted from the 18 networks are represented by rectangles on a wheel. ROIs are color coded according to the 18 networks. Group-level maps of the 18 functional networks are shown on the cortical surface outside the wheel. ROIs derived from the 18 networks could be grouped according to 7 well-studied canonical networks. The 25 ROI pairs that are most predictive of gF are indicated by thick lines in the wheel. Connections positively correlated with gF scores are shown in red, and connections negatively correlated with gF scores are shown in blue. Regions involved in these predictive connections are also plotted on the brain surface (bottom row). Warm colors indicate positive correlations between connectivity and gF. Cold colors indicate negative correlations between connectivity and gF. (**C**) The correlation between the predicted and observed gF scores was weaker (*p* = 0.002, *z* = 2.849, Steiger’s *z* test) when connectivity was estimated using the atlas-based ROIs (Pearson’s correlation, *r* = 0.207, *p* = 0.028). Correlation significance was determined using 1,000 permutations. (**D**) Twenty-six atlas-based ROI pairs that are most predictive of gF are plotted. See S1 Data for numerical values. ATN, attention; DMN, default mode network; FPN, frontoparietal network; gF, fluid intelligence; LMB, limbic; MOT, motor-sensory network; ROI, region of interest; SAL, salience network; VIS, visual.

Functional regions identified in individuals may capture the idiosyncrasies of subjects and lead to the discovery of meaningful imaging biomarkers for cognitive functions and behavior. Here, we explored the possibility of predicting individual subjects’ levels of fluid intelligence (gF) based on connectivity among the individually specified ROIs. A support vector regression (SVR) algorithm combining the leave-one-family-out cross validation (LOFOCV) was employed for the prediction (see Materials and methods). A variety of potential confounds, including sex, age, age^2^, sex*age, sex*age^2^, head size, overall head motion, and acquisition date, were regressed from both the imaging measures and the gF scores before the prediction. The prediction analysis was first carried out using functional connectivity values among the individually specified ROIs derived from our method. The predicted gF scores showed a significant correlation with the observed gF (Fig 5A, *r* = 0.303, *p* < 0.001, permutation test accounted for the family structure, 1,000 permutations). Connections that were most predictive of gF involved ROIs in the frontoparietal network (FPN), salience network (SAL), default mode network (DMN), and motor-sensory network (MOT) (Fig 5B). Specifically, higher gF appeared to be associated with stronger connectivity strength between FPN and some other networks, including the DMN, SAL, and MOT. In contrast, the correlation between the predicted and observed gF was reduced (*p* = 0.002, *z* = 2.849, Steiger’s *z* test) when the model was trained using connectivity among the 92 corresponding ROIs defined in Yeo’s atlas [32] (Fig 5C, *r =* 0.207, *p =* 0.028, permutation test accounted for the family structure, 1,000 permutations). More importantly, the predictive connections identified using these two approaches were largely different (Fig 5B and Fig 5D) and only showed small overlap (Dice’s coefficient = 0.25, see S4A Fig). To better understand why atlas-based connectivity became less predictive of gF compared with individually specified connections, we directly examined the correlation between gF and connectivity values among ROIs. Correlations between gF and these predictive connections derived from individual ROIs were significantly stronger (*p* < 0.001, *t* = 4.49, paired *t* test, see S4B Fig) than correlations between gF and the same connections defined using group-level ROIs. This result indicated that brain-behavior correlation was already obscured by the group-level atlas before the prediction model was applied, thus impairing the prediction of gF. Repeated analysis using another group-level atlas provided by Glasser and colleagues [22], which consisted of 360 ROIs, yielded similar results (correlation between predicted and observed gF: *r =* 0.215, *p =* 0.041, permutation test accounted for the family structure, 1,000 permutations, see S5 Fig).

### Size, position, and functional connectivity of the individually specified ROIs provide complementary information for predicting gF

**Fig 6:**
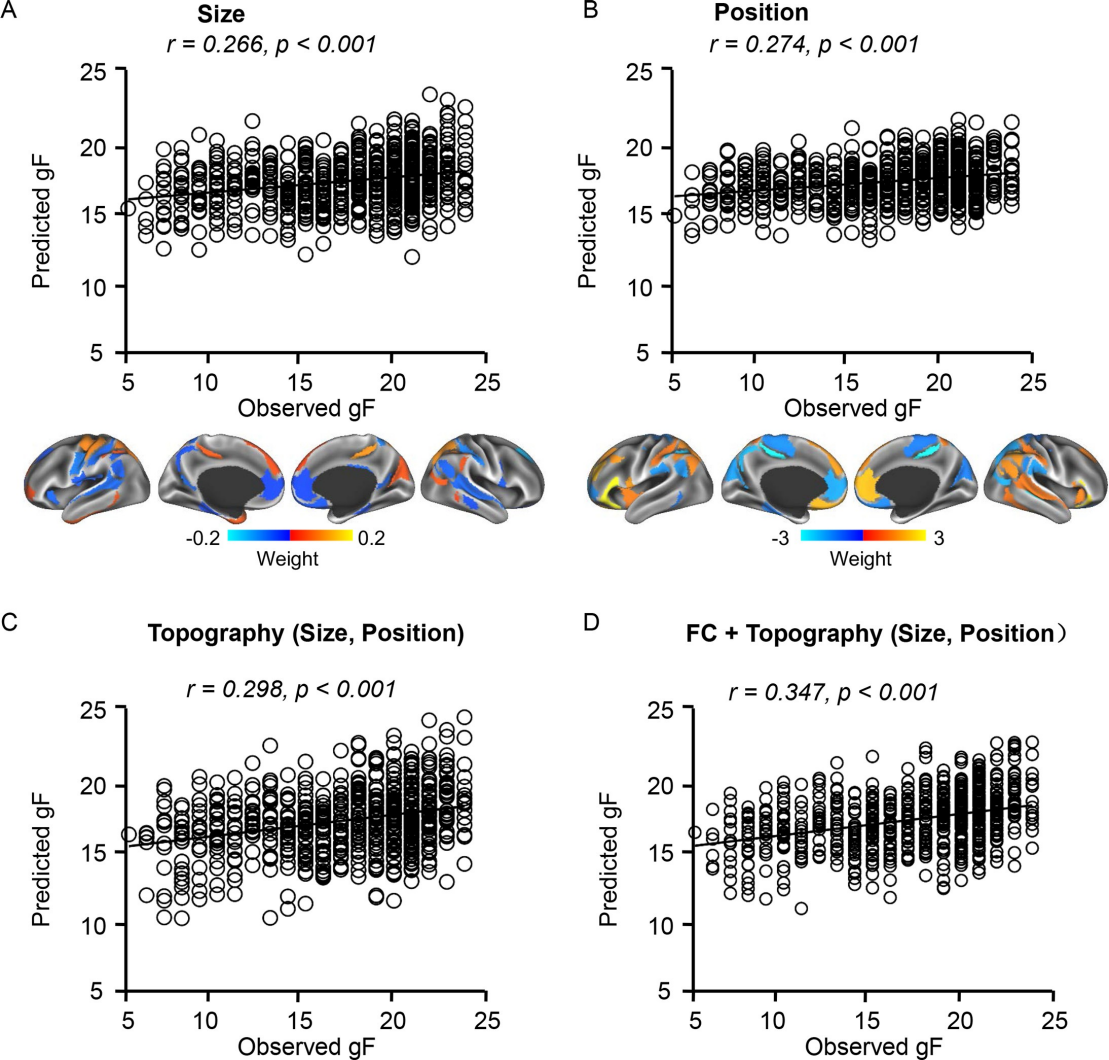
Topography of individually specified ROIs can predict gF. (A) Size of brain regions can predict gF. Regions with prediction weight values above the global mean were mapped on the surface. Warm colors indicated regions whose size showed positive correlations with gF scores. Cold colors indicated regions whose size showed negative correlations with gF scores. (B) Position of brain regions can predict gF. Brain regions whose coordinates on the anterior-posterior axis could predict gF were plotted on the brain surface. Warm color indicates that more anterior position is related to higher gF. Cold color indicates that more posterior position is related to higher gF. (C, D) Combining topography and functional connectivity features can improve the prediction of gF. See S1 Data for numerical values. gF, fluid intelligence; ROI, region of interest.

Our analysis above indicated that intersubject variability in the size, position, and connectivity of the functional regions can be dissociated using the individually specified ROIs (Fig 2). Here, we examined whether the topographic features (size and position) of the functional regions are behaviorally relevant. We found that the size and position of the individually specified ROIs could also predict gF scores (Fig 6, *r* = 0.266, *p < 0*.*001* for size; *r* = 0.274, *p* < 0.001 for position; *r* = 0.298, *p* < 0.001 for size and position combined; permutation test accounted for the family structure, 1,000 permutations). Specifically, we observed a mild negative correlation (*r* = −0.125, *p* = 0.001) between gF and size of the DMN regions (network 15 and 16, see S7 Fig for the network labels) but a positive correlation between gF and the size of the motor-sensory regions (*r* = 0.095, *p* = 0.013). Additionally, higher gF may be related to a more anterior position of a functional region (network 17) in the inferior frontal gyrus, which is likely related to language (*r* = 0.099, *p* = 0.010). Using functional connectivity and topography features together, the correlation between the predicted and observed gF increased to *r* = 0.347 (*p <* 0.001, permutation test accounted for the family structure, 1,000 permutations). These results indicated that the size, position, and connectivity of the functional ROIs provide nonredundant information for the prediction of behavior.

For comparison, we repeated the prediction analysis using ROIs defined in group-level atlases and data aligned by MSMAll [34]. After this multimodal alignment, functional connectivity strength among the atlas-based ROIs could better predict gF compared with connectivity derived from the unaligned data (*r =* 0.255 for Yeo’s atlas with MSMAll versus *r* = 0.207 for Yeo’s atlas without MSMAll; *r* = 0.300 for Glasser’s atlas with MSMAll versus *r* = 0.215 for Glasser’s atlas without MSMAll; see S5A Fig). Specifically, Glasser’s atlas combined with data aligned using MSMAll could predict gF with an accuracy comparable to that based on connectivity strength among the individualized ROIs. However, because the size and position of an individual subject’s brain regions cannot be specifically examined after MSMAll alignment, this atlas-based strategy (*r* = 0.300) did not outperform our individualized approach (*r* = 0.347), which takes advantage of individual differences in both connectivity strength and network topography.

Interestingly, we also found a negative correlation between gF and head motion (*r* = −0.122, *p* = 0.001 in the 677 subjects, see S6 Fig for analyses on the effect of head motion), consistent with previous reports [35]. The correlation increased when we included the subjects with greater head motion. In the 815 subjects who had completed resting state fMRI runs, the correlation between gF and head motion was *r* = −0.176 (*p* = 4.4 × 10^−7^). To investigate whether the prediction of gF was influenced by head motion, we calculated the partial correlation between predicted and observed gF, while controlling for head motion. We found that controlling for head motion had little effect on the correlations, indicating the correlations between observed and predicted behaviors were not driven by motion (S5B Fig). Finally, we repeated the prediction analysis based on the individual-specified features, using 10-fold cross validation, and found that our conclusions remained unchanged (correlation between predicted and observed gF was as follows: *r* = 0.295 for connectivity, *r* = 0.249 for ROI size, *r* = 0.270 for ROI position, see Materials and methods).

### Prediction of gF was largely driven by between-network connectivity

When inspecting the functional connections that were predictive of gF (as shown in Fig 5B), we found that the majority of them were connections between different functional networks rather than connections within the same network. In these predictive connections, many between-network connections appeared to be positively correlated with gF (red lines in Fig 5B), although some connections showed a negative correlation (blue lines in Fig 5B). To examine how between-network connectivity is related to gF, we averaged the connectivity values of all predictive between-network connections and found that mean between-network connectivity showed a mild positive correlation with gF (*r* = 0.131, *p* = 6.09 × 10^−4^), indicating that subjects with higher gF tend to have stronger between-network connectivity, especially between the FPN and several networks, including the DMN, SAL, and MOT. To understand why between-network connectivity derived from individually specified ROIs could better predict gF than that derived from atlas-based ROIs, we investigated how between-network connectivity was changed by the functional alignment. We found that the strength of between-network connectivity showed an average decrease of 12.07% when the ROIs were individually specified compared with group-level ROIs based on the Yeo atlas (S7 Fig, *p* < 0.001 for 16 of 18 networks, paired *t* test, Bonferroni correction for 18 comparisons). These findings indicated that between-network connectivity values were significantly overestimated in studies that directly applied the group-level atlas to individual subjects; thus, it must be interpreted with caution because the inflated connectivity values are more prone to type I errors [36]. Intriguingly, although the absolute values of between-network connectivity were significantly reduced after the functional alignment, they became more predictive of gF, suggesting that our individualized ROIs improved the specificity and accuracy of functional connectivity estimates.

## Discussion

Establishing proper functional correspondence between subjects is a prerequisite for group-level analyses of functional imaging measures. The present study explored the potential of conducting fMRI analyses based on a set of homologous regions identified in individuals. Taking advantage of a subject-level functional network parcellation technology, we were able to reliably identify the homologous functional regions across individuals. Intersubject variability in size, position, and connectivity of these functional regions was largely dissociated. We found that previously reported intersubject variability in vertex-wise functional connectivity maps was related to the variability in position of the functional regions. The homologous functional regions can be used to align task-fMRI maps across subjects and significantly improved group-level estimates of task-evoked activations in comparison with atlas-based alignment. Importantly, the individually specified ROIs were also able to capture the idiosyncrasies of subjects and better predicted individual differences in gF than atlas-based connectivity measures. We further demonstrated that not only the functional connectivity among ROIs but also the size and position of the ROIs are related to individual differences in human behavior. Collectively, these findings suggest that localizing functional regions in individual subjects can benefit a wide range of studies in the investigation of resting state functional connectivity, task activation, and brain-behavior associations.

### Identifying functional regions in individual subjects is essential for functional imaging research

Standard imaging processing procedures use volume-based [37,38] or surface-based [39,40] registration to align an individual subject’s functional data to a population-level brain template. These registration methods are based on anatomical features such as brain shape, curvature, sulcal depth, or their derivatives (e.g., spectral features of cortical anatomy) [41]. While they can align the macroanatomy of subjects to some extent, these approaches are not capable of aligning functional regions that are often dissociated from macroscopic anatomical landmarks. Recent progress in resting state functional connectivity research has made it possible to align data across subjects based on resting state networks and shows great promise in improving the estimate of connectivity and task-fMRI activations [28]. The functional alignment procedure aims to control for the “nuisance variance” introduced by the topography of networks; thus, one can accurately measure the connectivity strength or task-evoked activation across a group of subjects (but see Discussion below). In the present study, we showed that data alignment using subject-specific functional regions could significantly improve the group-level estimates of task activations (Fig 4) and functional connectivity, especially for the connections between different networks that tend to be overestimated by traditional methods (S7 Fig). The improved connectivity measures in turn can benefit the discovery of imaging biomarkers for cognitive abilities (Fig 5 and Fig 6).

Identifying functional regions in individual subjects not only improves task-fMRI and connectivity estimates, but also enables the investigation of intersubject variability in functional network topography (Fig 2). For example, we found that ROIs in association areas are highly variable in terms of their spatial distribution. In contrast, positions of the functional regions in the visual and auditory cortices are less variable across individuals, which is consistent with our previous knowledge that visual and auditory functions are more strongly tied to anatomical structures than association functions. However, after aligning the data based on homologous functional regions, intersubject variability in connectivity strength demonstrated an unexpected distribution (Fig 2D) and showed a high degree of intersubject variability in these primary functional areas. This implies that intersubject variability of visual and auditory functions may be mostly reflected in their connectivity strength with other brain regions. Further work is required to investigate how the connectivity strength variability in these areas may relate to individual differences in auditory and visual functions. This unexpected observation may lead to new testable hypotheses about individual differences in auditory and visual processing.

### Using functional regions derived from resting state connectivity for task-fMRI studies

An important question in the field of neuroimaging that has yet to be answered is whether resting state functional connectivity could serve as the “functional localizer” for task-fMRI analyses. Some previous studies have used simple fMRI tasks to localize functional ROIs in individual subjects prior to quantitative analyses of functional signals at the population level and have shown great potential in improving statistical power [42–45]. Nevertheless, functional mapping using task-based MRI at the single subject level generally suffers from poor signal-to-noise ratio (SNR), limited test-retest reliability [46–48], and inconsistency with respect to the current gold standard of functional mapping in individuals, i.e., invasive electrical cortical stimulation (ECS) [49,50]. Intrinsic functional connectivity may be an alternative, as it has demonstrated great strengths in individual-level functional mapping; however, understanding the exact relationship between intrinsic connectivity and task-evoked activation remains one of the key questions in brain imaging. At the population level, regions with strong intrinsic functional connectivity at rest tend to co-activate during tasks [51], indicating that spontaneous and task-evoked activity were bound by common functional configurations. In addition, the network architecture revealed by resting state connectivity is present across a wide variety of task states [52]. In line with these findings, we have previously demonstrated that at the single subject level, the whole-brain functional connectivity network architecture derived from task-fMRI data largely resembles that derived from resting state data [17]. Using a machine learning strategy, Tavor and colleagues recently showed the possibility of predicting individual subjects’ task-evoked activity based on combinations of resting state functional connectivity maps [53]. In the present study, we directly quantified the correlations between individual differences in cortical functional anatomy and individual differences in task-evoked activation patterns. The results indicate that spontaneous and task-evoked activity are tightly related to each other (Fig 3), supporting the possibility of using resting state connectivity as the functional localizer for task-fMRI analyses. We further showed that task-evoked activations were more robustly detected in the individually specified functional ROIs than in the atlas-based ROIs (Fig 4C and 4D). It was recently hypothesized that fMRI analyses based on the signals averaged within functional parcels might benefit from a “neurobiologically constrained” smoothing, which could improve the SNR and statistical power by avoiding the deleterious effects of spatial smoothing [22]. We found that task activations averaged within the individually specified ROIs were significantly more similar between individuals than activations averaged in the atlas-based ROIs (Fig 4B), thus suggesting that the improved statistical power can not only result from the neurobiologically constrained smoothing within subjects but also from the more accurate alignment of functional regions between subjects. Taken together, these data demonstrate the feasibility and advantages of using connectivity as the functional localizer for task-fMRI studies.

### Exploring brain-behavior associations based on individually specified functional regions

Recent evidence suggests that individual differences in human behavior and cognition, such as intelligence quotient, musical skills, and reading ability, may be related to variability in brain connectivity [54–60]. In a previous study, we carried out a meta-analysis and demonstrated that loci predicting individual differences in the behavioral and cognitive domains are predominantly located in the association cortex, including the language, executive control, and attention networks that are known to be wired more differently between individuals than the unimodal regions [5]. This observation implies that associations between functional connectivity estimates and behavioral measures may be underestimated or undetected if functional regions are not tailored to individual subjects. Previous studies mostly quantified functional connectivity within regions of population-based atlases, and then correlated these estimates with the individual subject’s behavioral and cognitive measures [59,61,62]. Here, we showed that performing analyses based on individually specified functional regions will improve the correspondence of functional connectivity and cognitive as well as behavioral measures, thereby facilitating the discovery of new imaging biomarkers for cognition and behavior. Importantly, we found that between-network connectivity measurement will greatly benefit from the subject-specific ROIs. Although the absolute values of between-network connectivity were significantly reduced after functional alignment, they became more predictive of gF. We observed that individuals with higher gF tend to show stronger between-network connectivity, especially between the FPN and several networks, including DMN, SAL, and MOT. Moreover, accurate qualification of between-network connectivity based on individualized ROIs will have particularly strong implications for clinical research, as recent studies have suggested that changes in between-network connectivity may signify normal brain development [63] as well as pathological changes [64]. The analytical framework developed in this study can be conveniently extended to the investigations of brain-behavior associations in clinical populations [65,66].

### Position and size of the functional regions are behaviorally relevant

A particularly important finding of this study is that not only the functional connectivity but also the size and position of the functional regions are related to gF (Fig 6), which is also known to be substantially heritable [67]. Our observations are in line with two recent studies that both stressed the importance of network topography. Bijsterbosch and colleagues [68] examined how individual differences in topographic features may influence the modelling of brain connectivity and demonstrated that the spatial arrangement of functional regions could predict nonimaging measures of behavior and lifestyle. By comparing the spatial topography of 17 networks across subjects, Kong and colleagues [69] also showed evidence that individual differences in large-scale network topography could predict individual differences in multiple behavioral phenotypes across cognition, personality, and emotion. While our results are consistent with these recent findings, the present study has proposed a framework that allows one to investigate the brain-behavior association for each discrete functional region and specifically examine imaging features that are largely dissociable, including the size, position, and connectivity of each region.

The significance of network topography in human brain function and behavior has been indicated in numerous studies (e.g., [70]) but has not been systematically investigated at the whole-brain level until recently. In our previous study, we observed that the sizes of some functional networks demonstrated strong hemispheric lateralization, which was also related to handedness and task-fMRI activation [17]. Here, we not only showed that individual differences in network topography are associated with individual differences in task activation patterns (Fig 3) but also showed that they are behaviorally relevant (Fig 6). These observations strongly suggest that variance in size and position should not be treated as nuisance variance and simply removed by the alignment procedure. Finally, given that gF is a heritable trait, future research can take advantage of our parcellation approach and specifically investigate whether the size, position, and connectivity strength of functional regions are influenced by different genetic factors.

### Clinical relevance of performing functional analyses based on individually specified regions

Remarkable progress and exciting discoveries have been made in the field of functional imaging research over the past two decades; however, only few of them have been directly connected to clinical interventions. A critical bottleneck for expanding the clinical use of fMRI is the ability to robustly localize functional circuits relevant to disorders in individual patients. For example, previous work using functional connectivity to identify potential biomarkers of neurological [71–74] and psychiatric [75–77] illnesses has repeatedly found evidence for altered network architecture in patients, as compared with healthy control participants. And yet, such group-level observations have failed to yield any biomarkers that can predict treatment response or provide confirmatory evidence of a patient's current symptoms and diagnosis. To meet clinical demands, a marker must reliably reflect a patient's current or future symptom load in a manner that can be applied to the management of individual patients [78]. Here, we demonstrated that individually specified functional regions can improve the detection of associations between imaging measures and cognitive abilities at the group level (Fig 5 and Fig 6), implying that this approach may facilitate the identification of neural circuits associated with symptom severity in patients. Critically, this subject-specific strategy may not only help to identify the symptom-related circuits but, at the same time, can map these circuits onto the individual patient’s brain, thus providing personalized targets for intervention.

### Limitations and caveats

There are several limitations related to the methodology employed in the present study. First, the functional ROIs were derived from a connectivity network parcellation, which uses a “winner-takes-all” approach. However, the resting state of the human brain is not a single static state, but consists of multiple states that dynamically emerge and dissolve. Functional network parcellation based on connectivity should thus be seen as a statistical estimate of the co-activation probability among brain regions, as opposed to a collection of fundamental functional units separated by sharp boundaries. Second, the functional regions identified in individual subjects depend on the validity of the network parcellation. The present study is based on the 18-network parcellation that has been widely used in the literature [32]. However, the optimal number of networks is yet to be investigated and will most likely remain equivocal. Moreover, the 18-network parcellation cannot reveal fine-grained subdivisions of important areas such as the auditory and visual cortices. Taking this crude parcellation as the basis for constructing individual-level parcels will inevitably limit its usage in the investigation of highly specialized functions within some areas. Third, performing group-level analyses using the subject-specific ROIs relies on the identification of homologous regions across subjects. Because the functional localization problem is inherently ambiguous, the procedure of matching homologous functional regions across individuals may introduce error or bias. For example, although the individualized parcellation approach may be able to segment the hemispheres of people with left-lateralized and right-lateralized language dominance differently, in extreme cases when the language area is missing in one hemisphere, it may not improve “true” functional alignment relative to a group atlas because it would not match one subject’s right-lateralized language region to another subject’s left-lateralized one. Fourth, the present study only focused on cortical regions; it did not include functional regions in subcortical structures. The involvement of cortico-subcortical circuits in various cognitive processes and brain disorders is well recognized. Future work on functional network parcellation in individual subjects’ subcortical structures will greatly advance our ability to characterize functional brain architecture. Finally, the reliability of functional ROIs is also dependent on the scan length. Recent studies, including our own [16,79], have provided evidence that sufficient scan length is crucial for the individual-level test-retest reliability of functional connectivity measurements. The relatively low test-retest reliability of the individualized parcellation boundaries in several brain regions may introduce noise relative to group atlases, which are defined with many more data than the data collected for a single subject. Whether functional ROIs derived from short resting state scans can benefit group-level functional analyses must be further explored.

## Materials and methods

### Ethics statement

The present study used data made publicly available by the HCP, supported by the WU-Minn Consortium. Written informed consent was obtained from each participant in accordance with relevant guidelines and regulations approved by the local institutional review board at Washington University in St. Louis (IRB #201204036).

### Participants and data acquisition

The present study used data from the HCP S900 data release, which consisted of 955 young healthy subjects. After quality control, 677 subjects (372 female, age range 22–35 years, except for one subject who was over 36 years) were selected for subsequent analyses. Each participant underwent two fMRI sessions on two different days. Each fMRI session consisted of two 15-minute resting state runs and about 30 minutes of task-fMRI. A battery of behavioral tests was performed by each participant. The present study examined the association between gF and neuroimaging measures. gF was selected because its association with functional connectivity has been reported in previous studies [54,59,80,81]. gF is one’s capacity to solve problems in novel situations.

More details about the participants and data acquisition can be found in S1 Text.

### Data processing

The “ICA-FIX” denoised fMRI data of the HCP subjects, represented as time series of grayordinates [28], were used. The data were already preprocessed in the HCP pipeline using FSL (FMRIB Software Library), FreeSurfer, and Connectome Workbench’s command line functions [28,31,82,83]. Each subject's preprocessed resting state fMRI data were resampled to a common standard cortical surface mesh representation (fs_LR 32k mesh). Studies have reported that global physiological noise and motion-related artifacts were not fully removed by ICA-FIX method [28,84]. We took the following additional processing procedures for resting state fMRI analysis: (1) normalizing the resting state fMRI time series at each vertex to zero mean and unit variance; (2) linear detrending and band-pass filtering (0.01–0.08 Hz); (3) regressing out 12 head-motion parameters and whole-brain signal; and (4) smoothing on the 32k fs_LR surface using a Gaussian smoothing kernel (sigma = 2.55 mm). Task-fMRI data were already preprocessed and analyzed by the HCP on the fs_LR 32k surface [31,85]. Task activation maps with 4-mm Gaussian smoothing were downloaded from the HCP, and we did not perform any additional processing on the task-fMRI data. For the group-level analyses on task activations shown in Fig 4, we computed the mean beta values in our individually specified ROIs, as well as in the ROIs defined by the atlas. Here, we used beta values (task effect size) instead of Z values (a ratio between beta and unexplained variance); thus, we could estimate the BOLD signal changes induced by tasks within a parcel [22]. The significance levels were estimated for each ROI using a one-sample *t* test.

For comparison purposes, we included resting state and task data that were processed using MSMAll, which is the improved intersubject registration based on a MSM algorithm and features from multiple imaging modalities released by the HCP [28,34]. Except where noted, the description of analysis applies to data aligned using the traditional cortical folding-based registration method.

### Population-level functional atlas

A population-level functional atlas including 18 cortical networks was obtained using data from 1,000 healthy subjects [17,32]. The original atlas consisted of 17 networks and was further divided into 114 discontinuous ROIs. The hand sensorimotor areas were then defined using a hand motor task and separated from other regions [86], resulting in 116 ROIs in total. Vertices at the boundaries of the ROIs were excluded because of the indefinite network affiliations. These population-level cortical ROIs were used as the functional template, and the homologous ROIs were identified in each individual subject.

### Identifying functional ROIs in individuals

The procedure to localize functional ROIs in individual subjects consisted of the following steps:

Step 1. Cortical functional networks were mapped in individual subjects using the iterative parcellation approach described in our previous work [17]. The algorithm was initially guided by the group-level functional network atlas derived from 1,000 healthy subjects. However, the influence of the atlas on the individual brain parcellation was not identical for every subject or every brain region and was thus flexibly adjusted based on the known distribution of interindividual variability and the SNR distribution in a particular subject. The influence of the population-based information gradually decreased as the iteration proceeded, allowing the final map to be completely driven by the individual subject’s data. The details of the iterative functional parcellation algorithm are described in Wang and colleagues [17].
Step 2. The cortical networks of individual subjects derived from Step 1 were segmented into discrete “patches” using a clustering algorithm (wb_command “metric-find-clusters” in the Connectome Workbench). To minimize the impact of noise and the matching costs, each cortical network on the surface was spatially smoothed using a Gaussian kernel function (sigma = 1 mm). The smoothing only affected the template matching procedure described below. Once a homologous ROI was recognized, the original unsmoothed region was used for subsequent analyses.
Step 3. Discrete patches in individual subjects were matched to the 116 cortical ROIs extracted from the population-level atlas that was used to guide the search of an individual subject’s networks. The template matching procedure was performed for each cortical network, ensuring that ROIs from one network will not be matched to ROIs from a different network. Here, our assumption is that a group-level ROI should roughly represent the center of the homologous ROIs from different individuals. Thus, to match ROIs across individuals, we use the group-level ROI as the common reference. If a subject-specific ROI falls within a certain distance from (not necessarily overlapping with) a group ROI of the same network, then it is reasonable to assume that the subject-specific ROI is corresponding to the group ROI. Because each network only has very few ROIs (usually one or two) in each lobe, this “finding the nearest neighbor” approach can very efficiently match ROIs across individuals. The detailed procedure is as follows: (1) if an individual-level patch overlapped (more than 20 vertices) with a single ROI in the group-level network, then the patch was labeled as the same ROI in the atlas. (2) If an individual-level patch overlapped with more than one ROI in the network, then the patch was split into multiple smaller patches. Specifically, vertices overlapping with the group-level ROIs were labeled according to these ROIs, forming the centers of several smaller patches. The remaining vertices in the original patch were then assigned to the nearest ROIs according to the geodesic distance on the brain surface. (3) If a patch did not overlap with any group-level ROI, then the patch was assigned to its nearest ROI if the shortest distance between the patch and the ROI was within a certain threshold; otherwise, the patch was labeled as “unrecognized”. In our algorithm, this threshold was selected as the mean distance between any two vertices in the nearest ROI.

### Calculating functional connectivity among ROIs

The individual subject’s connectivity profile was represented by the connectivity strength among ROIs. The mean signal of an ROI was computed by averaging the preprocessed BOLD signal across all vertices within the ROI. Connectivity between two ROIs was then estimated using Pearson’s correlation and converted into Z values using Fisher’s Z transformation. When ROIs were individually specified, we used the 92 ROIs that were consistently detected in all 677 HCP subjects; as a result, each individual subject’s connectome was represented by a 92 × 92 matrix.

For comparison purposes, functional connectivity was also estimated using the corresponding 92 ROIs in the population-level functional atlas [32] (“Yeo’s atlas”) and Yeo’s atlas with fMRI data aligned using MSMAll [34] (“Yeo’s atlas with MSMAll”). We applied the ROIs from Yeo’s atlas in the main analysis to ensure the number of ROIs was consistent with our individualized ROIs. In another comparison, 360 ROIs in a fine-grained atlas derived from the multimodal parcellation of the cortex proposed by Glasser and colleagues [22] (“Glasser’s atlas”) and the corresponding ROIs with fMRI data aligned using MSMAll (“Glasser’s atlas with MSMAll”) [22] were applied.

### Estimating within-network and between-network functional connectivity

Functional connections were separated into within-network and between-network connections according to whether they connected two ROIs in the same network or different networks (see S7 Fig). Within-network and between-network connectivity values were estimated for each subject. To compute the within-network connectivity of a specific network, we averaged the connectivity values of all ROI pairs within the network. To compute the between-network connectivity of a specific network, we averaged the connectivity values of all ROI pairs that involved an ROI within the network and an ROI outside the network.

### Estimating intersubject variability in size, position, and connectivity of the functional regions

The size of a functional region was calculated as the number of vertices that fell within that region. For each ROI, intersubject variability in size was calculated as the standard deviation of size across subjects. Intrasubject variability was estimated as the difference in ROI size between two scan sessions. To control for the impact of noise and other technical confounds on intersubject variability estimates, intersubject variability in size was corrected by regressing out the mean intrasubject variability using the similar strategy described in Mueller and colleagues [5]. The position of a functional region was represented by the coordinates of its center of mass. For each ROI, intersubject variability in ROI position was estimated as the average geodesic distance among the ROI centers across subjects. Intrasubject variability in ROI position was estimated as the geodesic distance between the ROI centers localized in two sessions. Intersubject variability in position was also corrected by regressing out the mean intrasubject variability. For a given ROI, intersubject variability in size and position was evaluated using only the subjects in whom the homologous ROIs could be detected.

Intersubject variability in vertex-wise and ROI-based functional connectivity was estimated using an approach similar to Mueller and colleagues [5]. For each vertex in the fsLR_32k surface mesh (59,412 vertices), the functional connectivity profile was represented by its connectivity with other vertices on the fsLR_32k surface. The functional connectivity profile for each individually specified ROI on the surface was computed as its connectivity with all other ROIs. Intersubject variability in ROI-based functional connectivity was also corrected by regressing out the mean intrasubject variability.

### Predicting gF using functional connectivity, size, and position of the functional ROIs

A SVR algorithm (L2-regularized L2-loss SVR model) implemented in the LIBLINEAR package (https://www.csie.ntu.edu.tw/~cjlin/liblinear/) was used to predict gF based on functional connectivity. Prediction performance was evaluated using LOFOCV. Family structure was kept intact in the prediction; i.e., subjects from the same family were not split into the training and testing datasets.

During the LOFOCV, model parameters were trained using the data of left-in subjects, and then the trained model was applied to the left-out subjects (i.e., one family) to predict the subjects’ gF scores; the procedure was repeated for each family to predict the gF score of all subjects. The performance of the prediction model was evaluated by the correlation between predicted and observed gF scores. Specifically, each LOFOCV procedure included feature selection, model learning, and testing. Before selecting effective features, covariates including sex, age, age^2^, sex*age, sex*age^2^, brain size, head motion, and acquisition quarter were regressed from the features and the observed gF scores. The regressing weights were applied to the left-out dataset.

To reduce redundant information and prevent possible over-fitting, functional connectivity that showed significant correlations with the gF scores in the training dataset were selected to train the model. We applied different significance thresholds (*p <* 0.001 and *p* < 0.0005) in feature selection, corresponding to large and small numbers of features. The de-confounded features from the testing data were inputted into the trained model to derive the predicted gF scores. To compare prediction performance based on different methods for defining functional ROIs or cross-subject alignment, the maximum correlation between predicted and observed gF was obtained from a different number of selected features.

To predict gF based on the size or position of the ROIs, we also used the SVR model described above. The position of a functional region on the cortical surface was evaluated as the coordinates of the region’s center of mass in the right–left axis, anterior–posterior axis, superior–inferior axis (RAS) coordinate system. In each LOFOCV, imaging features (i.e., size and/or position of ROIs) in the training data were applied to train the model. To test if topographic features from individually specified ROIs and functional connectivity among them can provide nonredundant information for the prediction of gF, we also trained the prediction models using different features and then averaged the outputs from different models.

The above prediction analyses based on connectivity, ROI size, and ROI position were also repeated using 10-fold cross validation. Specifically, we trained the model using 90% of the families and tested the model in the remaining 10% of the families. We ensured that family members were not split between folds. The 10-fold cross validation was repeated 50 times, and the mean prediction accuracy was reported.

To ensure that the prediction was not affected by head motion, we also investigated the relation between motion and gF scores, ROI size, and ROI reliability (See S6 Fig).

A nonparametric permutation test was performed to determine whether the prediction of gF scores exceeded the chance level. The observed gF values were randomly reshuffled among the subjects (1,000 permutations), and the prediction procedures were repeated. To account for family structure, members in one family were not split during the permutation [87]. The permutation *p*-value was estimated by calculating the percentage of permutations that yielded a prediction-observation correlation value higher than the prediction-observation correlation based on the real data. Contributions of the functional connections (connection weight) were averaged across all LOFOCV folds. If one feature was not selected in one fold, its contribution was set to zero in this fold. The contribution of a given ROI was calculated by summing up the contributions of all connections involving that ROI. If one ROI was not associated with any of the selected features, its contribution to the prediction was set to zero.

### Visualization

For the purpose of visualization, all imaging results were visualized using the Connectome Workbench display tool provided by the HCP (https://www.humanconnectome.org/) [83,88]. The connectograms in Fig 5B and Fig 5D were created using Circos (http://circos.ca/).

## Supporting information

S1 Text Participants and data acquisition. (DOCX)

S1 Data Numerical values underlie the summary data displayed in figures. (XLSX)

S1 Fig The procedure for identifying the homologous functional regions in individuals. This method utilized the following steps: (1) Cortical functional networks were mapped in individual subjects using an iterative parcellation approach [10]. (2) Each network was spatially smoothed and then segmented into multiple discrete patches. (3) A population-based atlas derived from a cortical network parcellation approach [11] was segmented into 116 discrete functional regions (ROIs). (4) Patches derived from each individual brain network were matched to the ROIs extracted from the same functional network in the population atlas. A patch may be matched to a single ROI or split to multiple smaller ROIs, and will be discarded when there is no matching ROI in the atlas. (5) Patches that matched the atlas-based ROIs were labeled as the homologous ROIs in the individual. ROI, region of interest.
(TIF)

S2 Fig Intersubject variability in vertex-wise functional connectivity is influenced by intersubject variability in topography and connectivity strength of the functional regions. (**A**) Intersubject variability in resting state functional connectivity quantified at each vertex was summarized in ROIs from Yeo’s atlas. (**B**) Intersubject variability in ROI size showed a moderate correlation (*r* = 0.26) with the variability in vertex-wise connectivity. (**C**) Intersubject variability in ROI position showed a strong correlation (*r* = 0.49) with the variability in vertex-wise connectivity. (**D**) Intersubject variability in connectivity among individually specified ROIs showed a strong correlation (*r* = 0.55) with the variability in vertex-wise connectivity. See S1 Data for numerical values. ROI, region of interest.
(TIF)

S3 Fig Task-activated regions detected in subsets of the cohort (*n* = 20, 30, 40, 50) and in the full cohort. (A) Between-subject similarity values during 4 tasks estimated by different approaches. (B) Group-level statistical analyses (one-sample *t* test) were performed for 4 tasks in the HCP data (Gambling, Relational, Social, and Emotional tasks) using the activation values in our individually specified ROIs or atlas-based ROIs. Regions with a significance value of *p* < 0.0001 (Bonferroni corrected for 92 comparisons) are displayed. (C) Group-level task-activated regions mapped in subsets of the subjects using a series of significance thresholds (logarithmic scale). See S1 Data for numerical values. ROI, region of interest.
(TIF)

S4 Fig (A) Functional connections that were predictive of gF scores from individual ROIs (red) and connections that were predictive of gF scores from group-level ROIs (blue) show moderate overlap (Dice’s coefficient = 0.25). (B) The same predictive connections showed weaker correlations (*p* < 0.001, paired *t* test) with gF when the connections were defined using the atlas (black) compared with connections defined in individuals (red). Each circle represents the correlation value between one predictive connection and gF. See S1 Data for numerical values. gF, fluid intelligence; ROI, region of interest.
(TIF)

S5 Fig (A) The bar plots show correlation between the observed gF and gF predicted using different approaches, including using ROIs in Yeo’s atlas [11], ROIs in Glasser’s atlas [12], Yeo’s atlas and Glasser’s atlas ROIs on data aligned by MSMAll [13], as well as our individually specified ROIs. (B) Controlling head motion had little effect on the prediction results. The bar plots show partial correlation values between predicted and observed gF, while controlling for motion. See S1 Data for numerical values. gF, fluid intelligence; MSM, multimodal surface matching; ROI, region of interest.
(TIF)

S6 Fig Head motion effects. (A) Pearson’s correlations between mean relative motion and node reliability. For each ROI, node reliability was calculated as the Dice’s overlap between the ROIs derived from the two scan sessions of the same subject. The map shows the uncorrected significance values (logarithmic scale) for the correlations between motion and node reliability. The reliability of two ROIs (indicated by the arrows in the map) was significantly correlated with motion. We used a significance threshold of *p* < 0.05 after Bonferroni correction (logarithmic scale: −log10(0.05/116) = 3.37). (B) Correlation between head motion and node size. No significant correlation was found between motion and node size. (C) Head motion effect on the similarity (Dice’s overlap) between the individualized ROI and atlas-based ROI (Yeo’s atlas). One ROI in the temporal pole (indicated by the arrow) was significantly affected by motion. (D) Negative correlation (*r* = −0.122, *p* = 0.001) was found between head motion and gF. Each subject is represented by a circle in the scatterplot. See S1 Data for numerical values. gF, fluid intelligence; ROI, region of interest.
(TIF)

S7 Fig Between-network connectivity was more accurately estimated using individually specified ROIs than using atlas-based ROIs. The strength of between-network connectivity showed an average decrease of 12.07% when the ROIs were individually specified compared with atlas based (*p* < 0.001 for 16 of 18 networks, paired *t* test, Bonferroni correction for 18 comparisons). The individualized functional ROIs were determined in each individual using data from the first scan session, while between-network connectivity values were estimated using data from the second scan session. See S1 Data for numerical values. ROI, region of interest.
(TIF)

## Abbreviations

DMN: default mode network
fMRI: functional MRI
FPN: frontoparietal network
gF: fluid intelligence
HCP: Human Connectome Project
LOFOCV: leave-one-family-out cross validation
MOT: motor-sensory network
MSM: multimodal surface matching
ROI: region of interest
SAL: salience network
SNR: signal-to-noise ratio
SVR: support vector regression
