# Supplementary material for: Performing group-level functional image analyses based on homologous functional regions mapped in individuals
Source: PLoS Biol. 2019 Mar 25;17(3):e2007032. doi: 10.1371/journal.pbio.2007032 (PMC6448916; doi:10.1371/journal.pbio.2007032)
Supplement: S1 Text — (DOCX) [file pbio.2007032.s001.docx]

**Participants and data acquisition**

The present study used data from the Human Connectome Project (HCP) S900 data releases, which consisted of 955 young healthy subjects. After quality control, 677 subjects (372 female, age range 22 – 35 years, except for one subject who was over 36 years) were selected for subsequent analyses based on the following inclusion criteria: 1) data of four resting state fMRI runs and seven task fMRI sessions are available; 2) each resting state fMRI run has 1200 time points; 3) mean relative head displacement of each resting state run is smaller than 0.15 mm [1]; 4) behavioral measurements and demographic information including fluid intelligence (gF, “PMAT24_A_CR”) and age are available in the “unrestricted” and “restricted” information spreadsheets. Written informed consent was obtained from each participant in accordance with relevant guidelines and regulations approved by the local institutional review board at Washington University in St. Louis (IRB # 201204036). Each participant underwent two fMRI sessions on two different days. Each fMRI session consisted of two 15 min resting-state runs (one run with left-to-right direction phase encoding and one run with right-to-left direction) and about 30 min of task-fMRI. Except where noted, the description of data processing and analysis applies to the two combined sessions. The tasks included working memory (WM), gambling (GAMBLING), motor (MOTOR), language (LANGUAGE), social cognition (SOCIAL), relational processing (RELATIONAL), and emotional processing (EMOTIONAL). MRI data were acquired on a 3-Tesla Siemens “Connectome Skyra” scanner with a 32 channel head coil. Structural T1 weighted and T2 weighted images were acquired with 0.7 mm isotropic resolution. Functional data were obtained using a gradient echo-planar pulse sequence (TR = 720 ms; TE = 33.1 ms; flip angle = 52°; FOV = 208 × 180 mm; slice thickness = 2.0 mm; 72 slices; 2 mm isotropic voxels, multiband factor = 8; echo spacing = 0.58 ms; bandwidth = 2290 Hz/Px; time points = 1200). To aid the coregistration of fMRI and structural data, a field map matched to the fMRI acquisition was also acquired. Detailed descriptions about the data set have been previously reported [2-4].

A battery of behavioral tests was performed by each participant. The present study examined the association between gF and neuroimaging measures. gF was selected because its association with functional connectivity has been reported in previous studies [5-8]. gF is one’s capacity to solve problems in novel situations. For each participant, gF was estimated using an abbreviated version of Raven’s Progressive Matrices [9]. Participants were asked to find the best-fitting square to fill a void in an arrangement pattern from a range of response choices. This task had 24 items and gF scores were recorded as the number of correct items. The data were made publicly available by the HCP (PMAT24_A_CR from <http://humanconnectome.org/data>).

**References**

1. Shen, X., et al., *Using connectome-based predictive modeling to predict individual behavior from brain connectivity.* Nat. Protocols, 2017. **12**(3): p. 506-518.

2. Van Essen, D.C., et al., *The WU-Minn Human Connectome Project: an overview.* Neuroimage, 2013. **80**: p. 62-79.

3. Van Essen, D.C., et al., *The Human Connectome Project: a data acquisition perspective.* Neuroimage, 2012. **62**(4): p. 2222-2231.

4. Barch, D.M., et al., *Function in the human connectome: task-fMRI and individual differences in behavior.* NeuroImage, 2013. **80**: p. 169-89.

5. Finn, E.S., et al., *Functional connectome fingerprinting: identifying individuals using patterns of brain connectivity.* Nat Neurosci, 2015. **18**(11): p. 1664-1671.

6. Cole, M.W., et al., *Global connectivity of prefrontal cortex predicts cognitive control and intelligence.* J Neurosci, 2012. **32**(26): p. 8988-99.

7. Santarnecchi, E., et al., *Efficiency of weak brain connections support general cognitive functioning.* Hum Brain Mapp, 2014. **35**(9): p. 4566-82.

8. Hearne, L.J., J.B. Mattingley, and L. Cocchi, *Functional brain networks related to individual differences in human intelligence at rest.* 2016. **6**: p. 32328.

9. Bilker, W.B., et al., *Development of abbreviated nine-item forms of the Raven's standard progressive matrices test.* Assessment, 2012. **19**(3): p. 354-69.
