# Supplementary figures and images for: Performing group-level functional image analyses based on homologous functional regions mapped in individuals
Source: PLoS Biol. 2019 Mar 25;17(3):e2007032. doi: 10.1371/journal.pbio.2007032 (PMC6448916; doi:10.1371/journal.pbio.2007032)

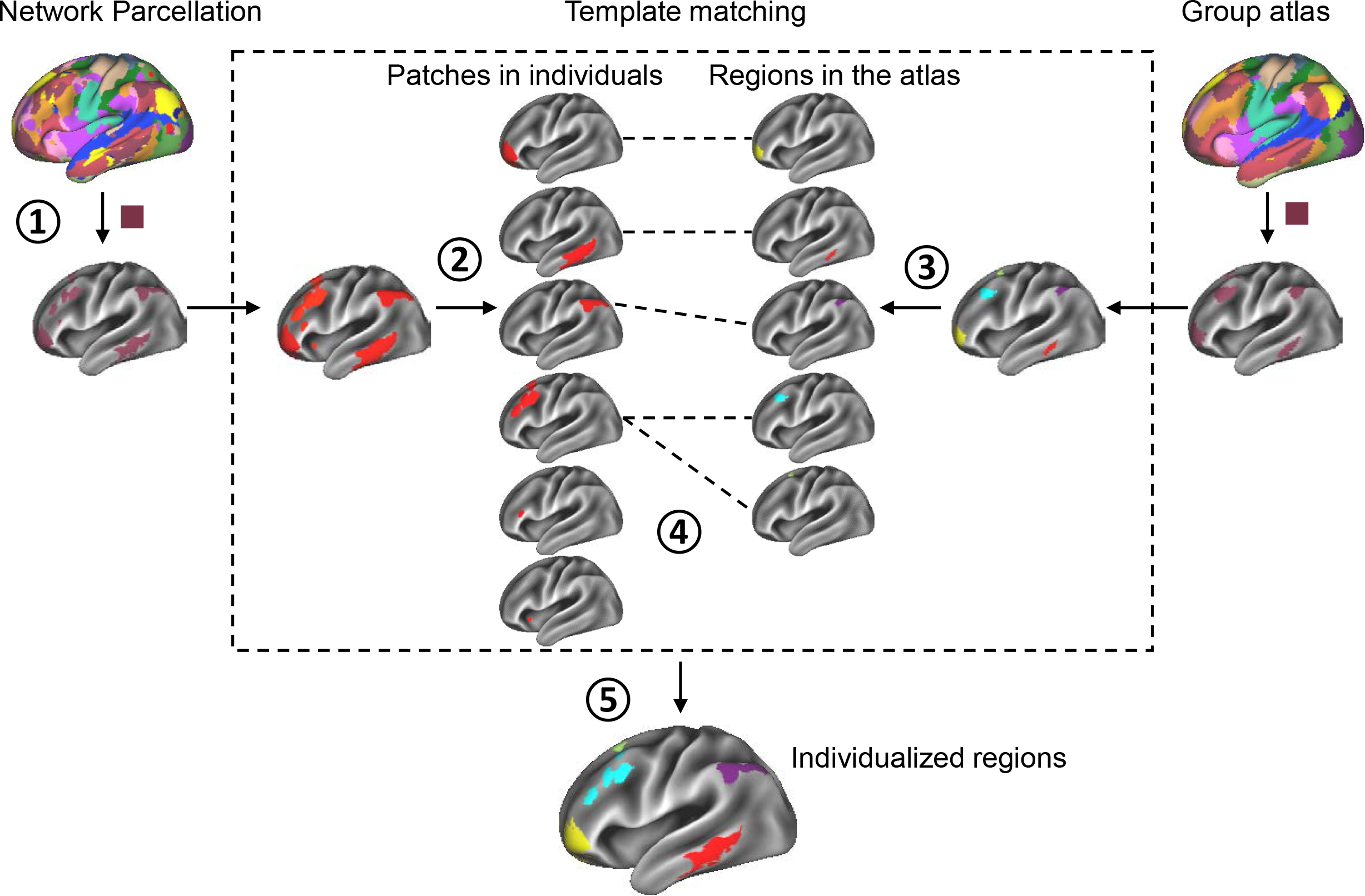

Supplement: S1 Fig — This method utilized the following steps: (1) Cortical functional networks were mapped in individual subjects using an iterative parcellation approach [10]. (2) Each network was spatially smoothed and then segmented into multiple discrete patches. (3) A population-based atlas derived from a cortical network parcellation approach [11] was segmented into 116 discrete functional regions (ROIs). (4) Patches derived from each individual brain network were matched to the ROIs extracted from the same functional network in the population atlas. A patch may be matched to a single ROI or split to multiple smaller ROIs, and will be discarded when there is no matching ROI in the atlas. (5) Patches that matched the atlas-based ROIs were labeled as the homologous ROIs in the individual. ROI, region of interest. (TIF) [file pbio.2007032.s003.tif]

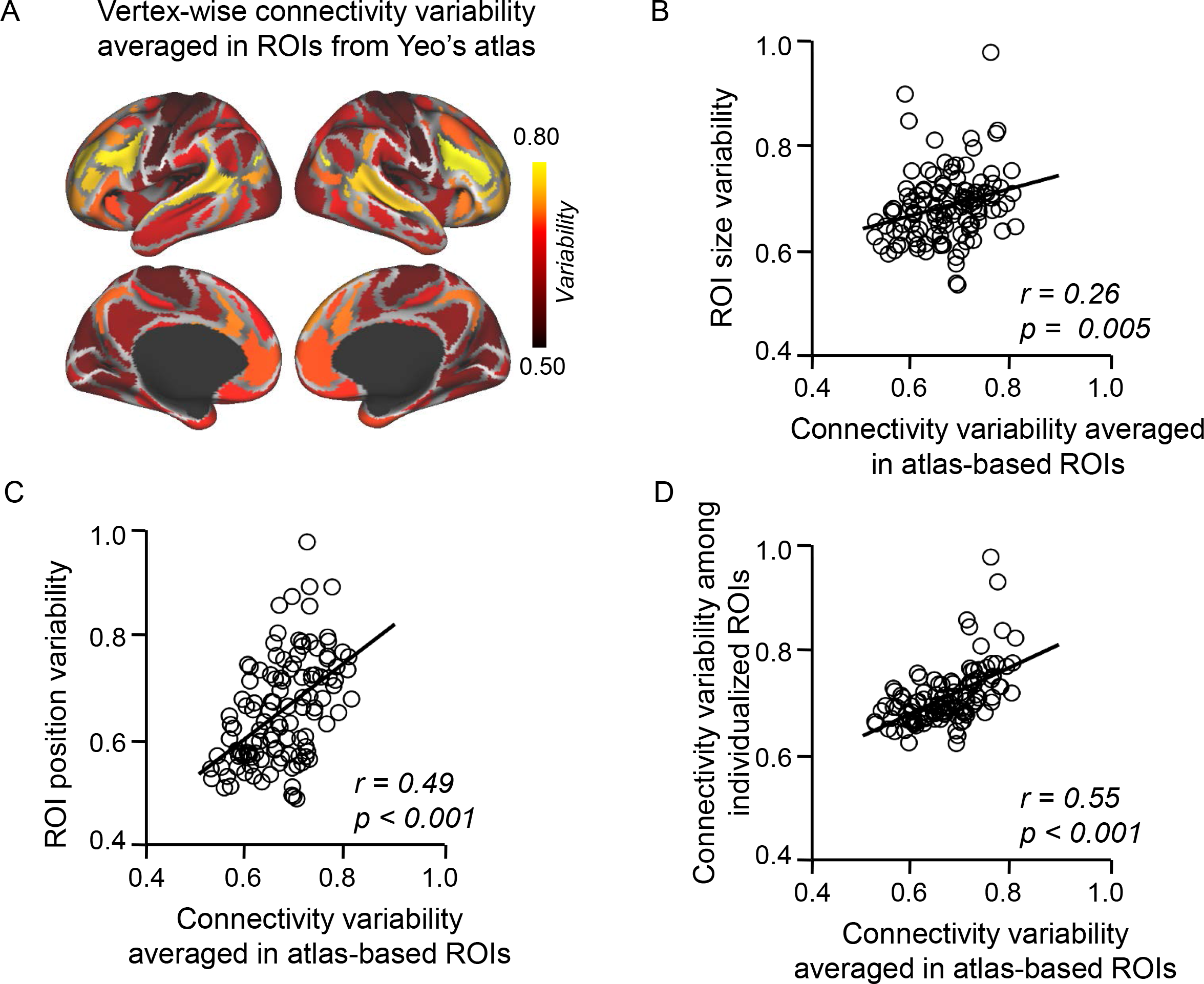

Supplement: S2 Fig — (A) Intersubject variability in resting state functional connectivity quantified at each vertex was summarized in ROIs from Yeo’s atlas. (B) Intersubject variability in ROI size showed a moderate correlation (r = 0.26) with the variability in vertex-wise connectivity. (C) Intersubject variability in ROI position showed a strong correlation (r = 0.49) with the variability in vertex-wise connectivity. (D) Intersubject variability in connectivity among individually specified ROIs showed a strong correlation (r = 0.55) with the variability in vertex-wise connectivity. See S1 Data for numerical values. ROI, region of interest. (TIF) [file pbio.2007032.s004.tif]

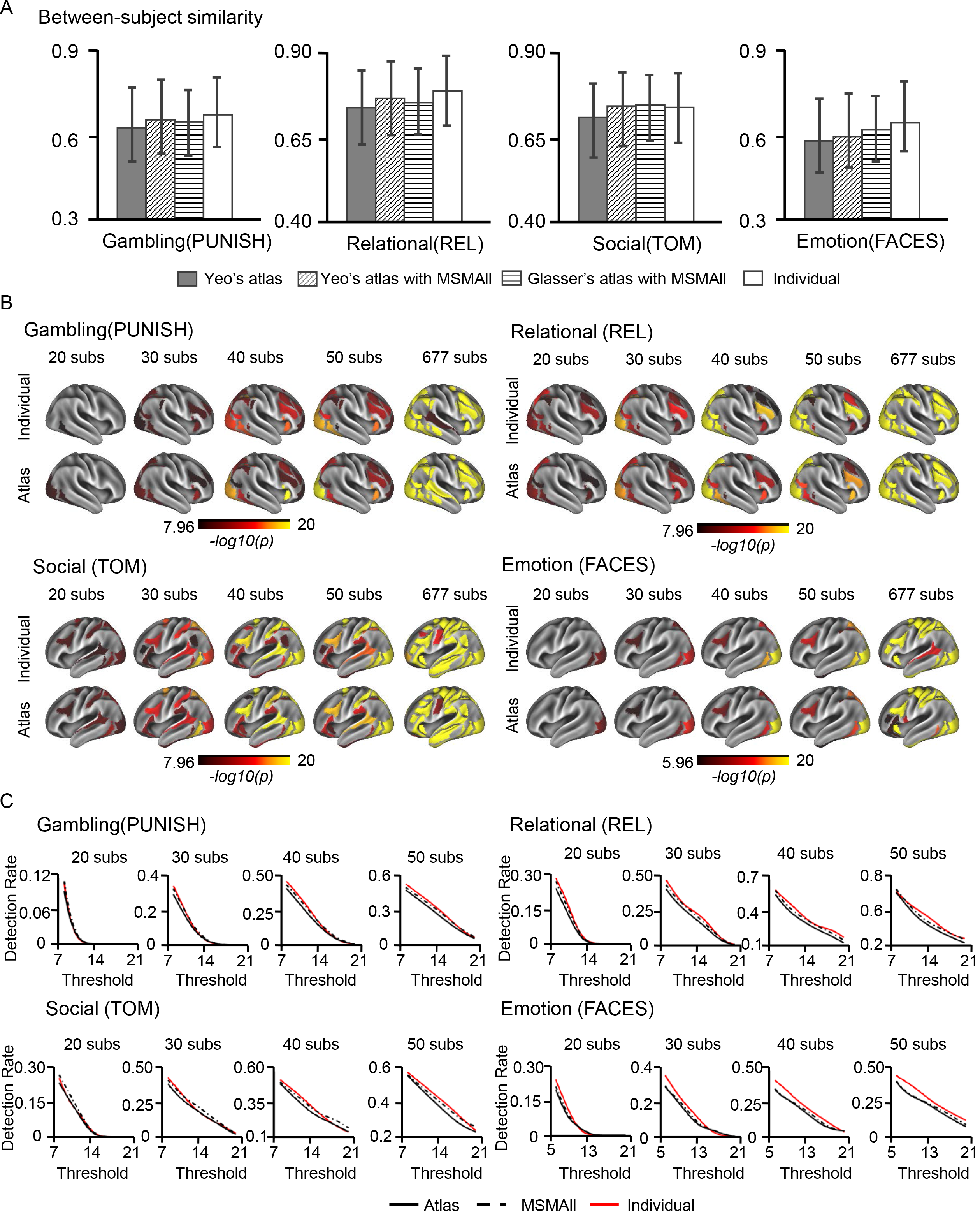

Supplement: S3 Fig — (A) Between-subject similarity values during 4 tasks estimated by different approaches. (B) Group-level statistical analyses (one-sample t test) were performed for 4 tasks in the HCP data (Gambling, Relational, Social, and Emotional tasks) using the activation values in our individually specified ROIs or atlas-based ROIs. Regions with a significance value of p < 0.0001 (Bonferroni corrected for 92 comparisons) are displayed. (C) Group-level task-activated regions mapped in subsets of the subjects using a series of significance thresholds (logarithmic scale). See S1 Data for numerical values. ROI, region of interest. (TIF) [file pbio.2007032.s005.tif]

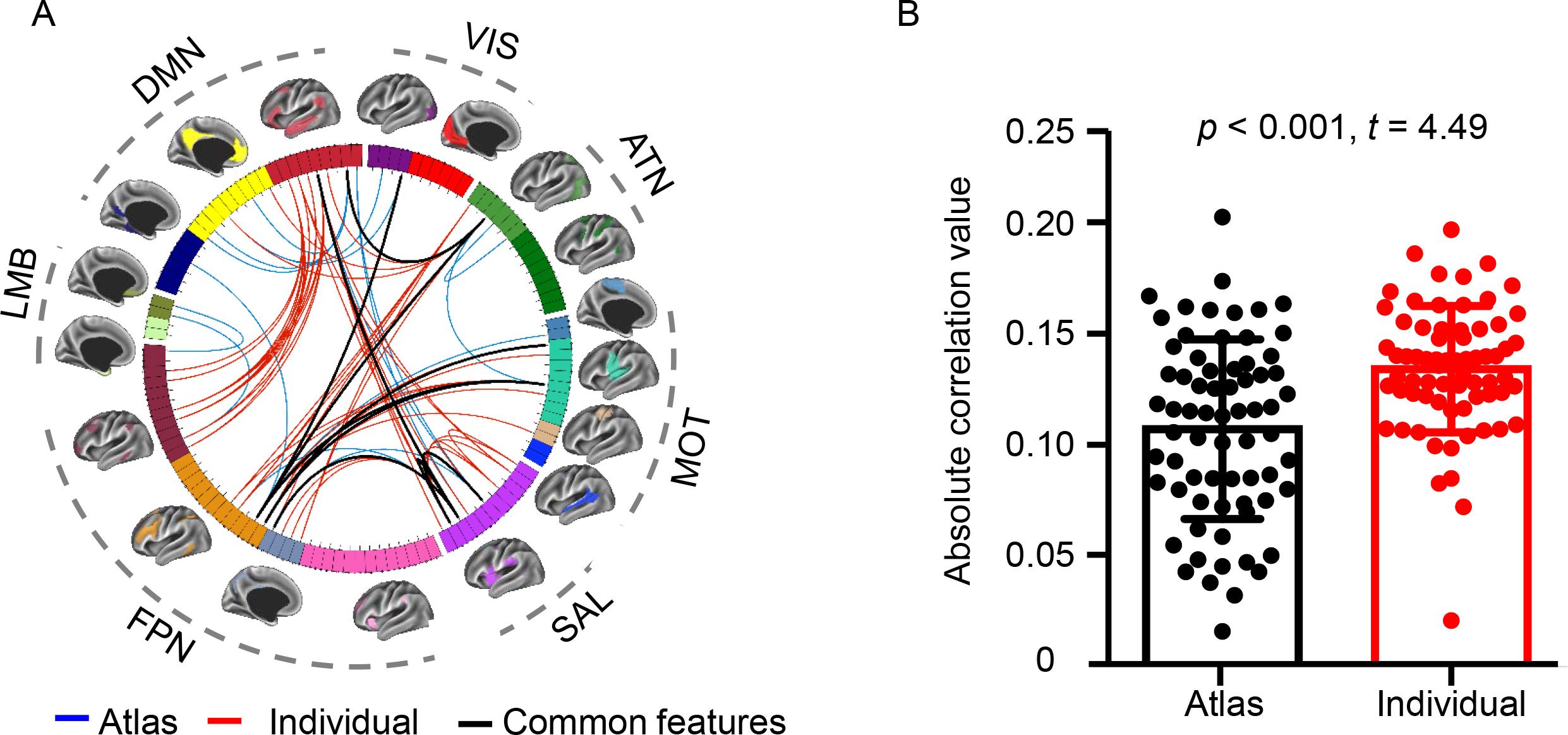

Supplement: S4 Fig — (A) Functional connections that were predictive of gF scores from individual ROIs (red) and connections that were predictive of gF scores from group-level ROIs (blue) show moderate overlap (Dice’s coefficient = 0.25). (B) The same predictive connections showed weaker correlations (p < 0.001, paired t test) with gF when the connections were defined using the atlas (black) compared with connections defined in individuals (red). Each circle represents the correlation value between one predictive connection and gF. See S1 Data for numerical values. gF, fluid intelligence; ROI, region of interest. (TIF) [file pbio.2007032.s006.tif]

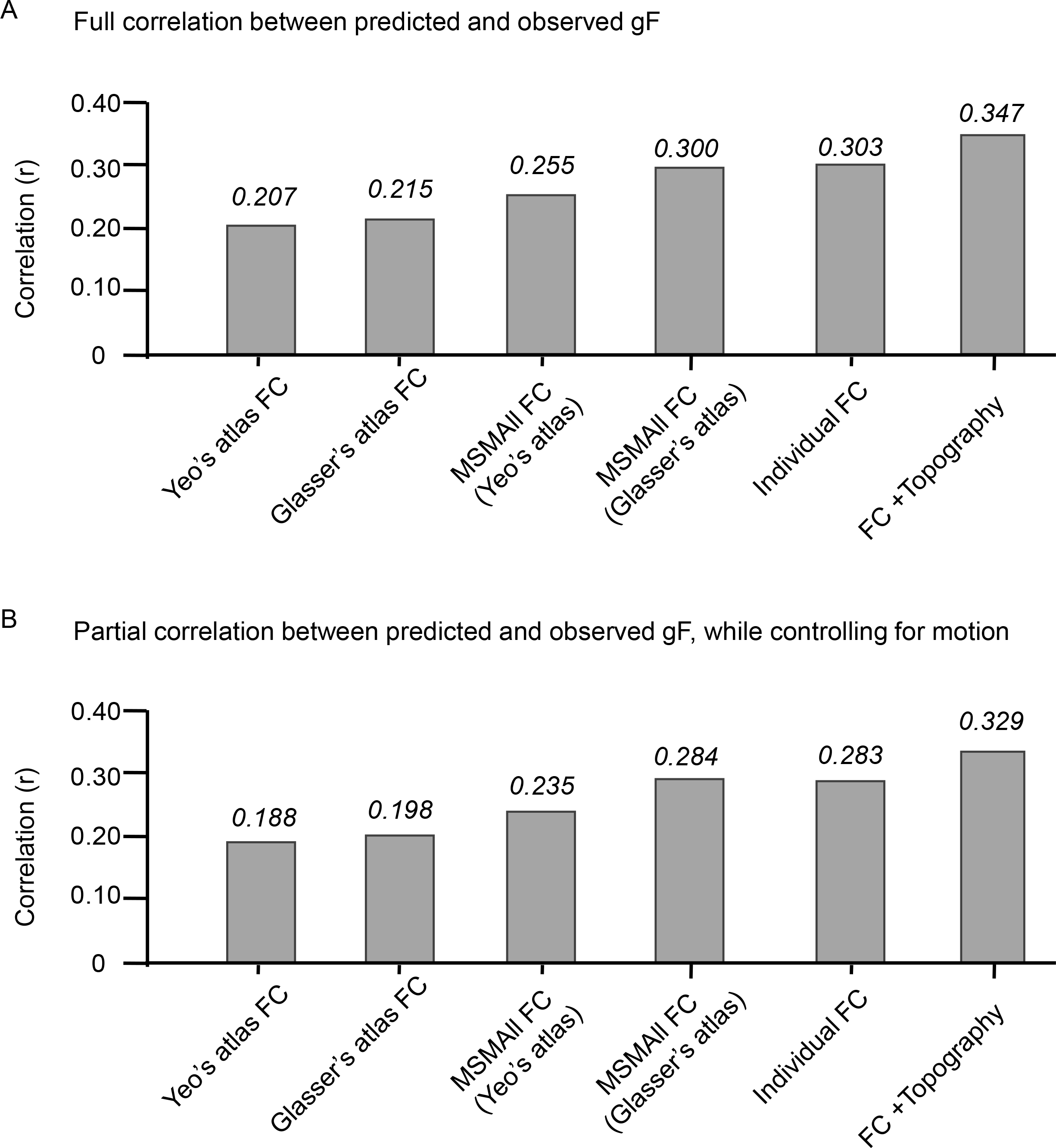

Supplement: S5 Fig — (A) The bar plots show correlation between the observed gF and gF predicted using different approaches, including using ROIs in Yeo’s atlas [11], ROIs in Glasser’s atlas [12], Yeo’s atlas and Glasser’s atlas ROIs on data aligned by MSMAll [13], as well as our individually specified ROIs. (B) Controlling head motion had little effect on the prediction results. The bar plots show partial correlation values between predicted and observed gF, while controlling for motion. See S1 Data for numerical values. gF, fluid intelligence; MSM, multimodal surface matching; ROI, region of interest. (TIF) [file pbio.2007032.s007.tif]

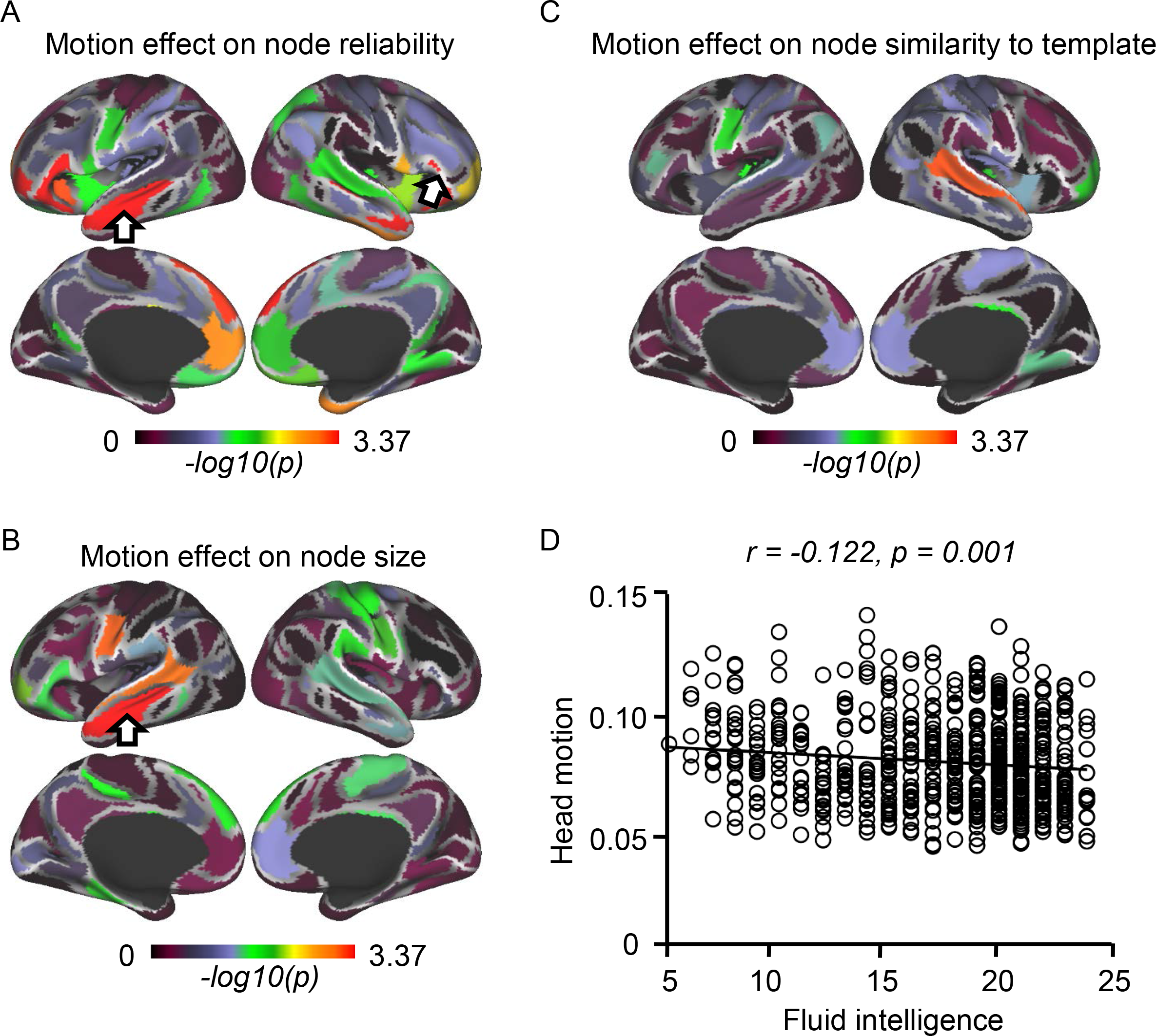

Supplement: S6 Fig — (A) Pearson’s correlations between mean relative motion and node reliability. For each ROI, node reliability was calculated as the Dice’s overlap between the ROIs derived from the two scan sessions of the same subject. The map shows the uncorrected significance values (logarithmic scale) for the correlations between motion and node reliability. The reliability of two ROIs (indicated by the arrows in the map) was significantly correlated with motion. We used a significance threshold of p < 0.05 after Bonferroni correction (logarithmic scale: −log10(0.05/116) = 3.37). (B) Correlation between head motion and node size. No significant correlation was found between motion and node size. (C) Head motion effect on the similarity (Dice’s overlap) between the individualized ROI and atlas-based ROI (Yeo’s atlas). One ROI in the temporal pole (indicated by the arrow) was significantly affected by motion. (D) Negative correlation (r = −0.122, p = 0.001) was found between head motion and gF. Each subject is represented by a circle in the scatterplot. See S1 Data for numerical values. gF, fluid intelligence; ROI, region of interest. (TIF) [file pbio.2007032.s008.tif]

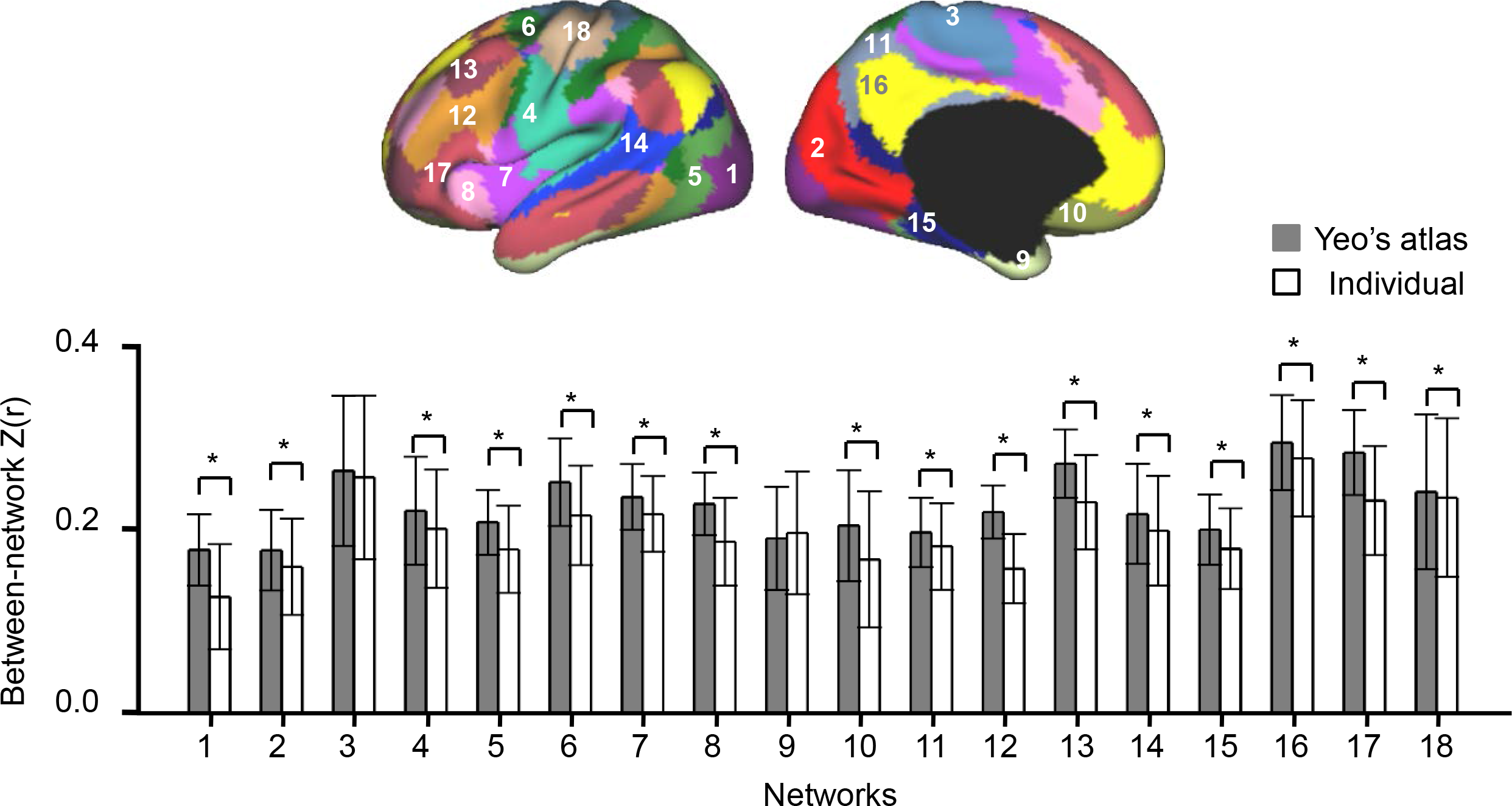

Supplement: S7 Fig — The strength of between-network connectivity showed an average decrease of 12.07% when the ROIs were individually specified compared with atlas based (p < 0.001 for 16 of 18 networks, paired t test, Bonferroni correction for 18 comparisons). The individualized functional ROIs were determined in each individual using data from the first scan session, while between-network connectivity values were estimated using data from the second scan session. See S1 Data for numerical values. ROI, region of interest. (TIF) [file pbio.2007032.s009.tif]
